# Supplementary material for: Nuc domain electrostatics drive the trans cleavage activity of CRISPR–Cas12a
Source: Nucleic Acids Res. 2026 Jan 9;54(1):gkaf1485. doi: 10.1093/nar/gkaf1485 (PMC12784958; doi:10.1093/nar/gkaf1485)
Supplement: gkaf1485_Supplemental_File [file gkaf1485_supplemental_file.pdf]

Supplementary Data

for

Nuc domain electrostatics drive the *trans* cleavage activity of CRISPR-Cas12a

Anthony Newman[1]*, Lora Starrs[1], Gaetan Burgio[1]*

[1] The Shine-Dalgarno Centre for RNA Innovation, Division of Genome Sciences and Cancer, The John Curtin School of Medical Research, The Australian National University, Canberra, ACT, 2601, Australia.

*To whom correspondence should be addressed.

Email: anthony.newman@anu.edu.au; gaetan.burgio@anu.edu.au

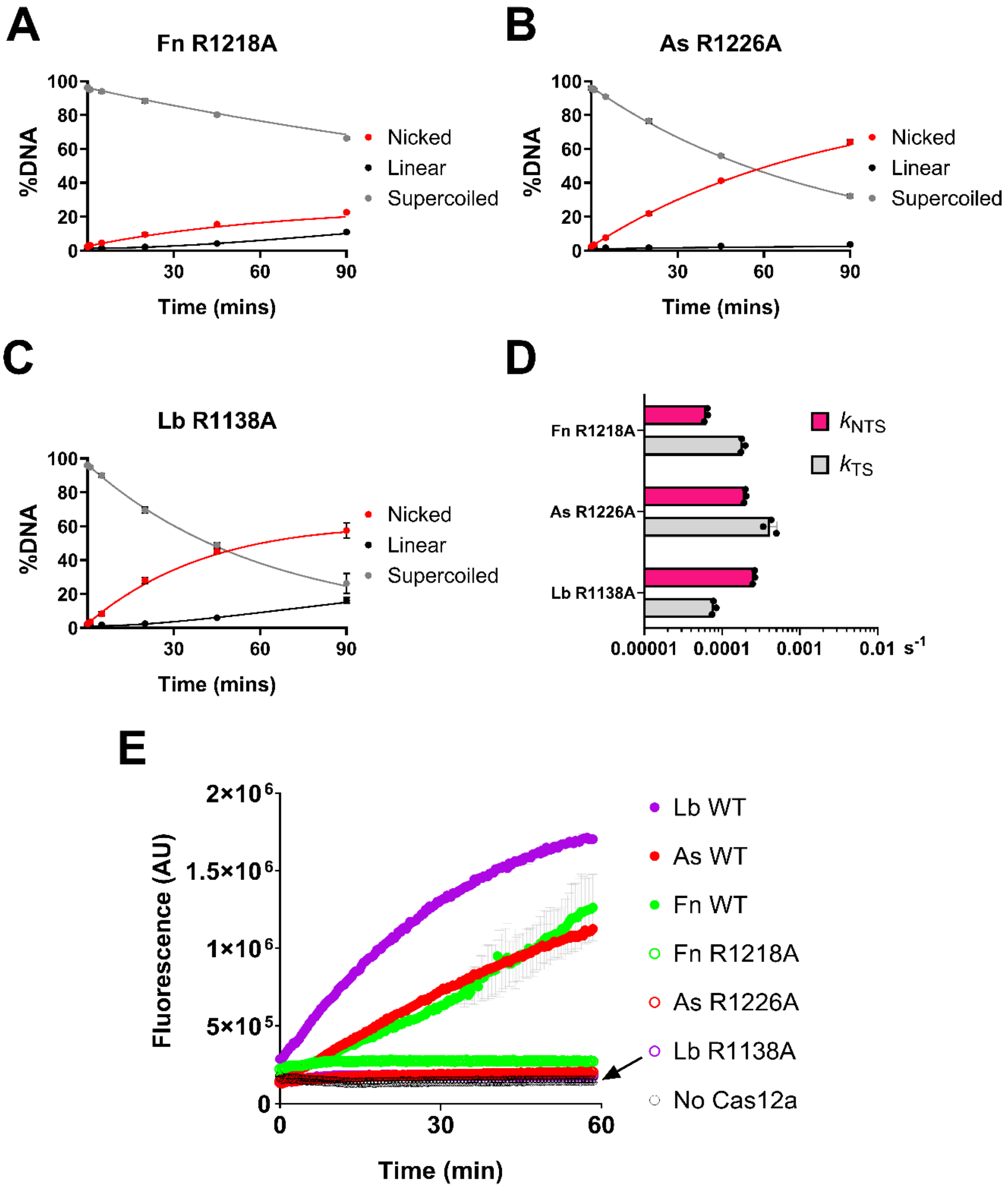

Figure S1: Time-course plasmid *cis* cleavage by (**A**) FnR1218A, (**B**), AsR1226A, and (**C**) LbR1138A. Dots show mean, error bars show s.d (not visible for FnR1218A and AsR1226A). Lines show fit of sequential NTS and TS cleavage model. (**D**) Kinetic values (s^{-1}) of NTS (k_{NTS}, pink) and TS cleavage (k_{TS}, grey), for the Cas12a indicated. Dots show individual replicates, bars show mean, error bars show s.d. (**E**) *Trans* cleavage of WT and mutants of FnCas12a, AsCas12a, and LbCas12a, when activated by 1 nM DNMT1 target dsDNA. Dots show mean, error bars show s.d.

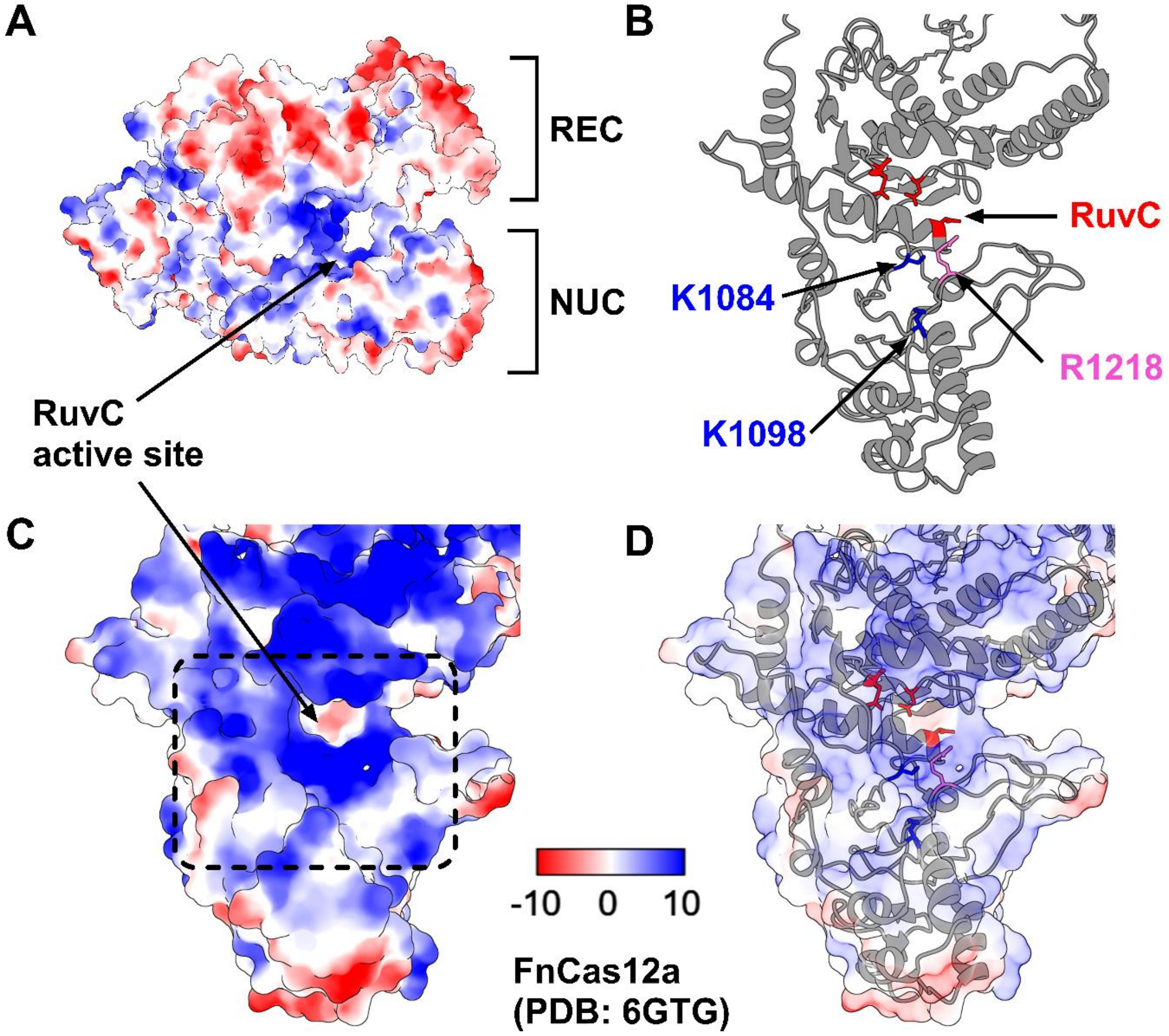

FigureS2: (**A**) Coulombic electrostatic potential of FnCas12a (PDB: 6GTG), as calculated by ChimeraX. Electrostatic potential is displayed on a colour gradient showing negative (red), neutral (white), and positive (blue). REC and NUC lobes are annotated, as is location of RuvC active site. (**B**) Ribbon representation of the NUC lobe, with key amino acids highlighted; RuvC active site residues (red), conserved adjacent arginine (pink), and positively charged residues subject to alanine substitution (blue). (**C**) Electrostatic potential of the NUC lobe, with arrow showing RuvC site. Dashed box shows cropped region shown in **Figure 1A** of main text. (**D**) Merge of ribbon representation with electrostatic potential (at 80% transparency).

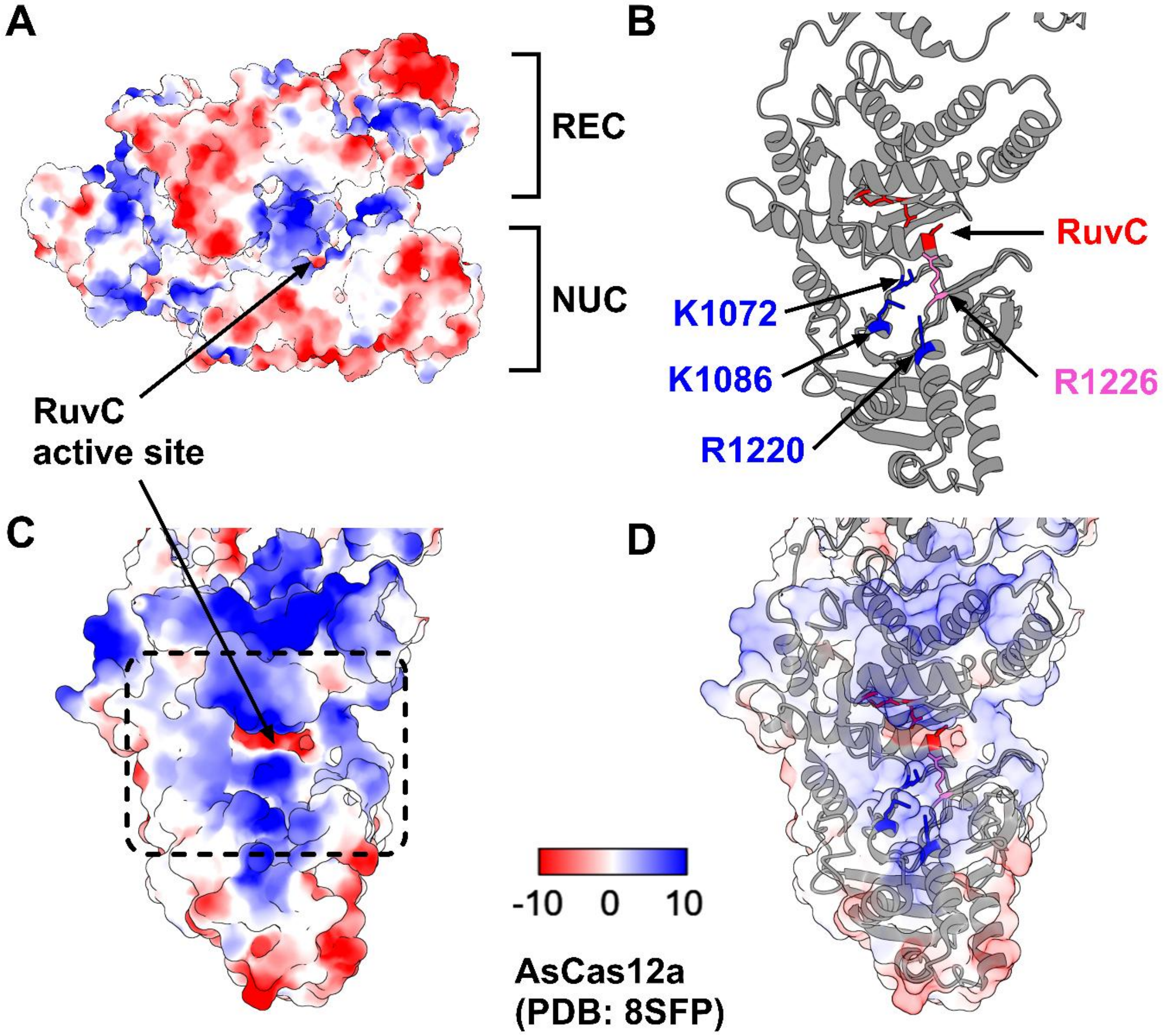

FigureS3: (**A**) Coulombic electrostatic potential of AsCas12a (PDB: 8SFP), as calculated by ChimeraX. Electrostatic potential is displayed on a colour gradient showing negative (red), neutral (white), and positive (blue). REC and NUC lobes are annotated, as is location of RuvC active site. (**B**) Ribbon representation of the NUC lobe, with key amino acids highlighted; RuvC active site residues (red), conserved adjacent arginine (pink), and positively charged residues subject to alanine substitution (blue). (**C**) Electrostatic potential of the NUC lobe, with arrow showing RuvC site. Dashed box shows cropped region shown in **Figure 1B** of main text. (**D**) Merge of ribbon representation with electrostatic potential (at 80% transparency).

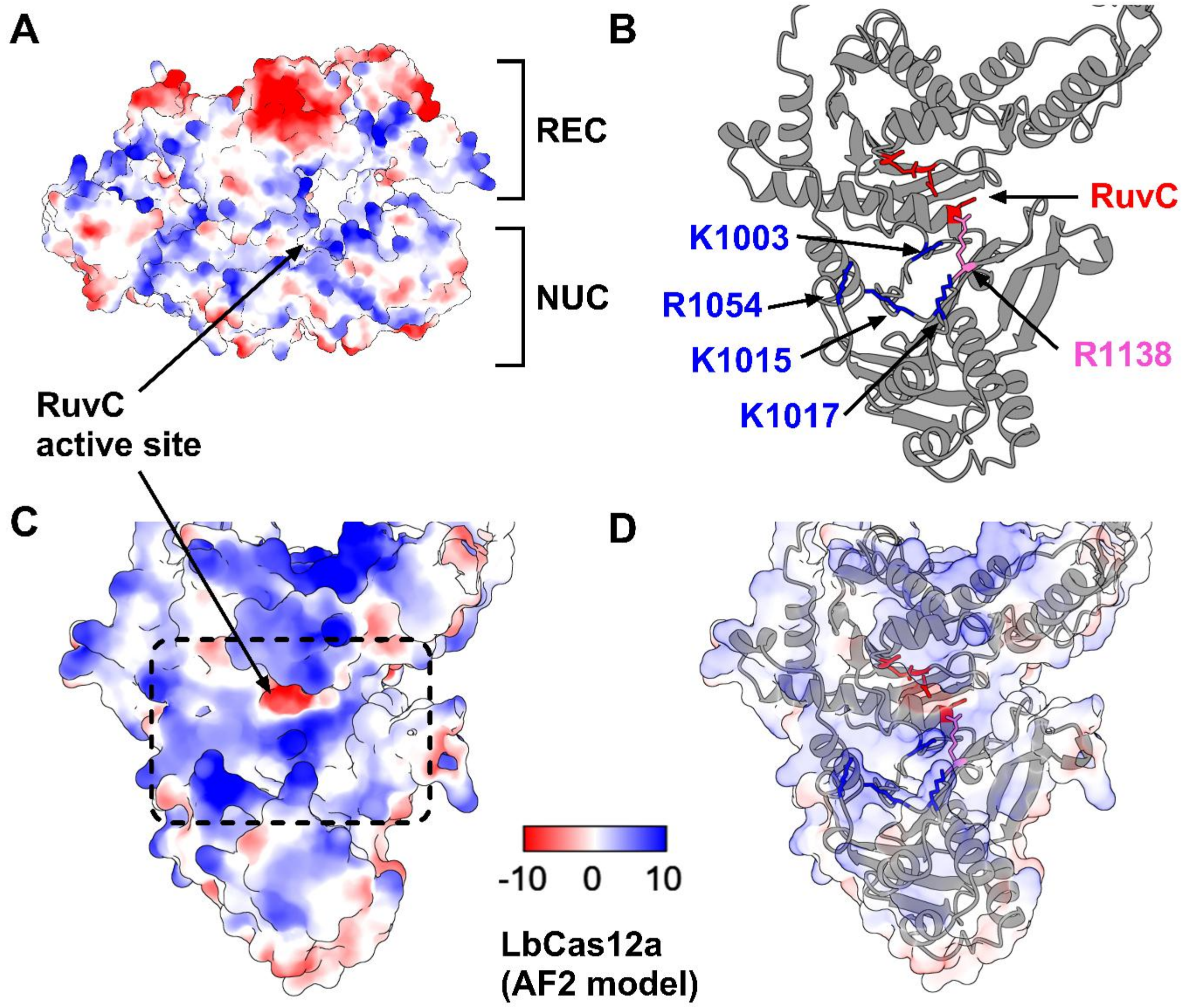

FigureS4: (**A**) Coulombic electrostatic potential of LbCas12a (AlphaFold monomer V2.0 prediction of A0A182DWE3), as calculated by ChimeraX. Electrostatic potential is displayed on a colour gradient showing negative (red), neutral (white), and positive (blue). REC and NUC lobes are annotated, as is location of RuvC active site. (**B**) Ribbon representation of the NUC lobe, with key amino acids highlighted; RuvC active site residues (red), conserved adjacent arginine (pink), and positively charged residues subject to alanine substitution (blue). (**C**) Electrostatic potential of the NUC lobe, with arrow showing RuvC site. Dashed box shows cropped region shown in **Figure 1C** of main text. (**D**) Merge of ribbon representation with electrostatic potential (at 80% transparency).

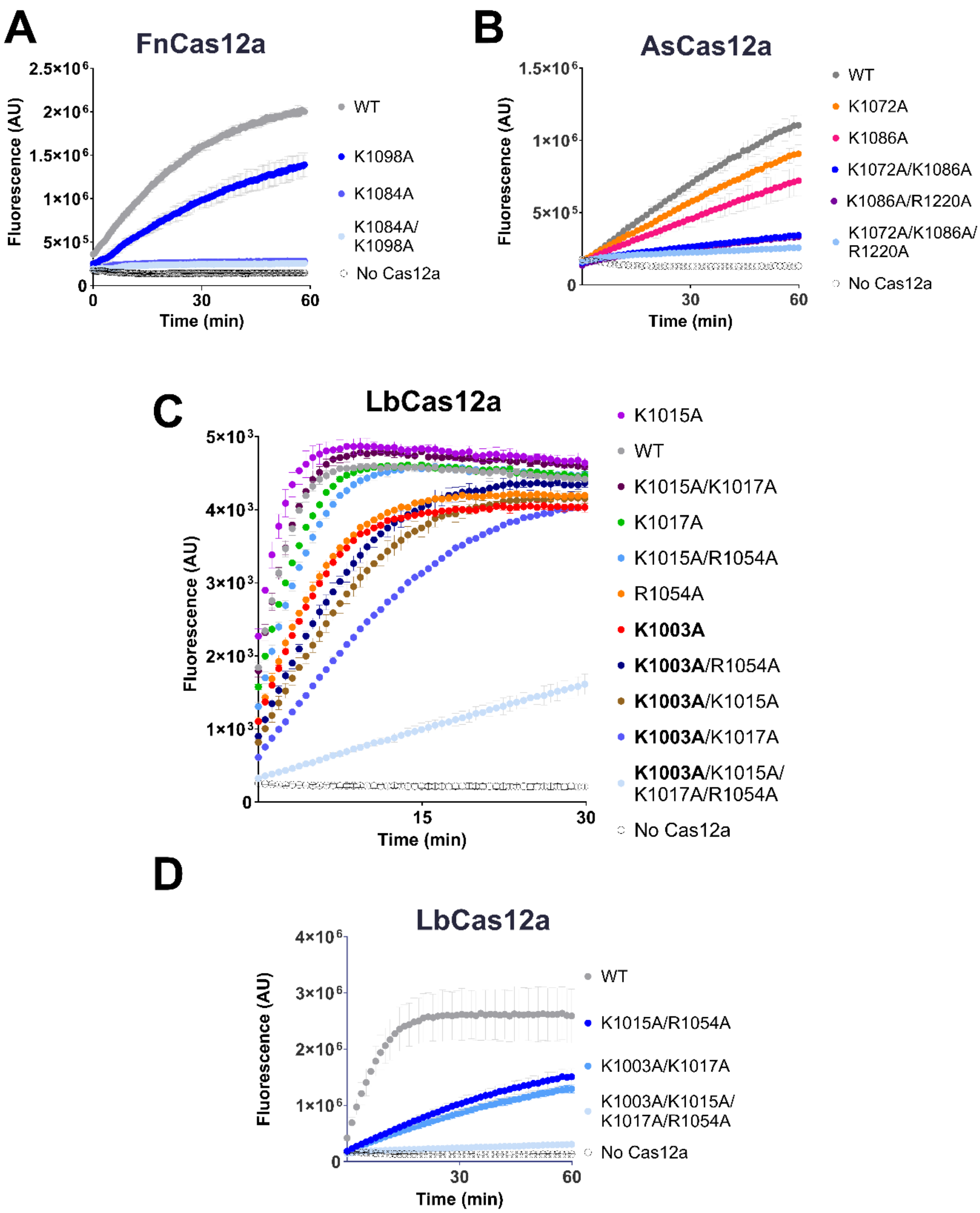

Figure S5: (**A**) *Trans* cleavage curve of WT FnCas12a and mutants, when activated by 1 nM DNMT1 dsDNA. (**B**) *Trans* cleavage curve of WT AsCas12a and mutants when activated by 1 nM DNMT1 dsDNA. (**C**) *Trans* cleavage curve of WT LbCas12a and mutants when activated by 5 nM DNMT1 target-strand ssDNA. Key mutation K1003A in bold. (**D**) *Trans* cleavage curve of WT LbCas12a and mutants, when activated by 1 nM DNMT1 dsDNA. Reaction buffers contained 50 mM NaCl (final concentration).

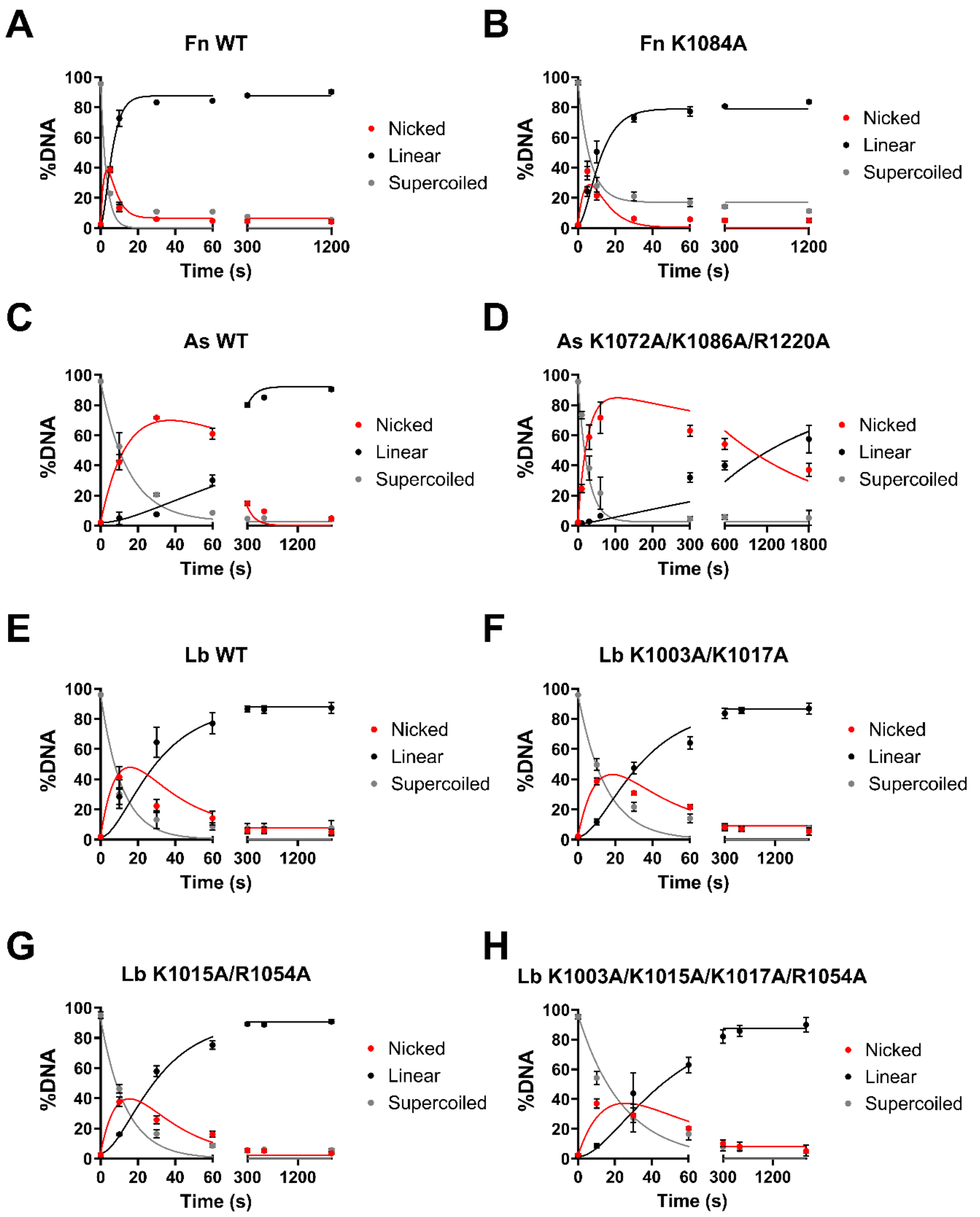

Figure S6: Time-course plasmid *cis* cleavage by (**A**) FnCas12a WT, (**B**) FnK1084A, (**C**) AsCas12a WT, (**D**) AsK1072A/K1086A/R1220A, (**E**) LbCas12a WT, (**F**) LbK1003A/K1017A, (**G**) LbK1015A/R1054A, (**H**) LbK1003A/K1015A/K1017A/R1054A. Dots show mean, error bars show s.d. Lines show fit of sequential NTS and TS cleavage model. Conducted in reaction buffers with a final concentration of 50 mM NaCl.

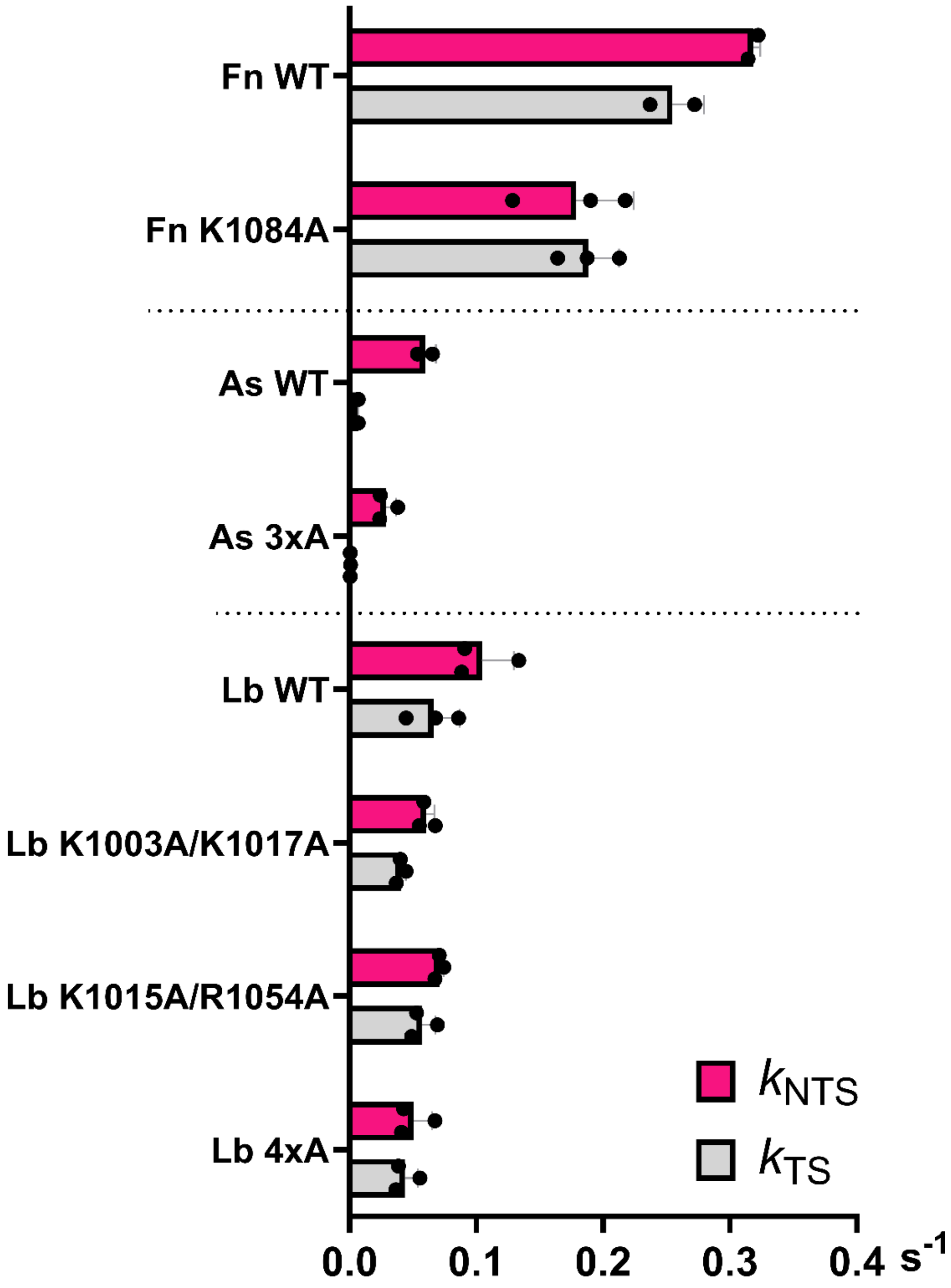

Figure S7: Kinetic values (s^{-1}) of NTS (k_{NTS}, pink) and TS cleavage (k_{TS}, grey), for the Cas12a indicated. Dots show individual replicates, bars show mean, error bars show s.d. Derived from **Figure S6** for the Cas12a indicated.

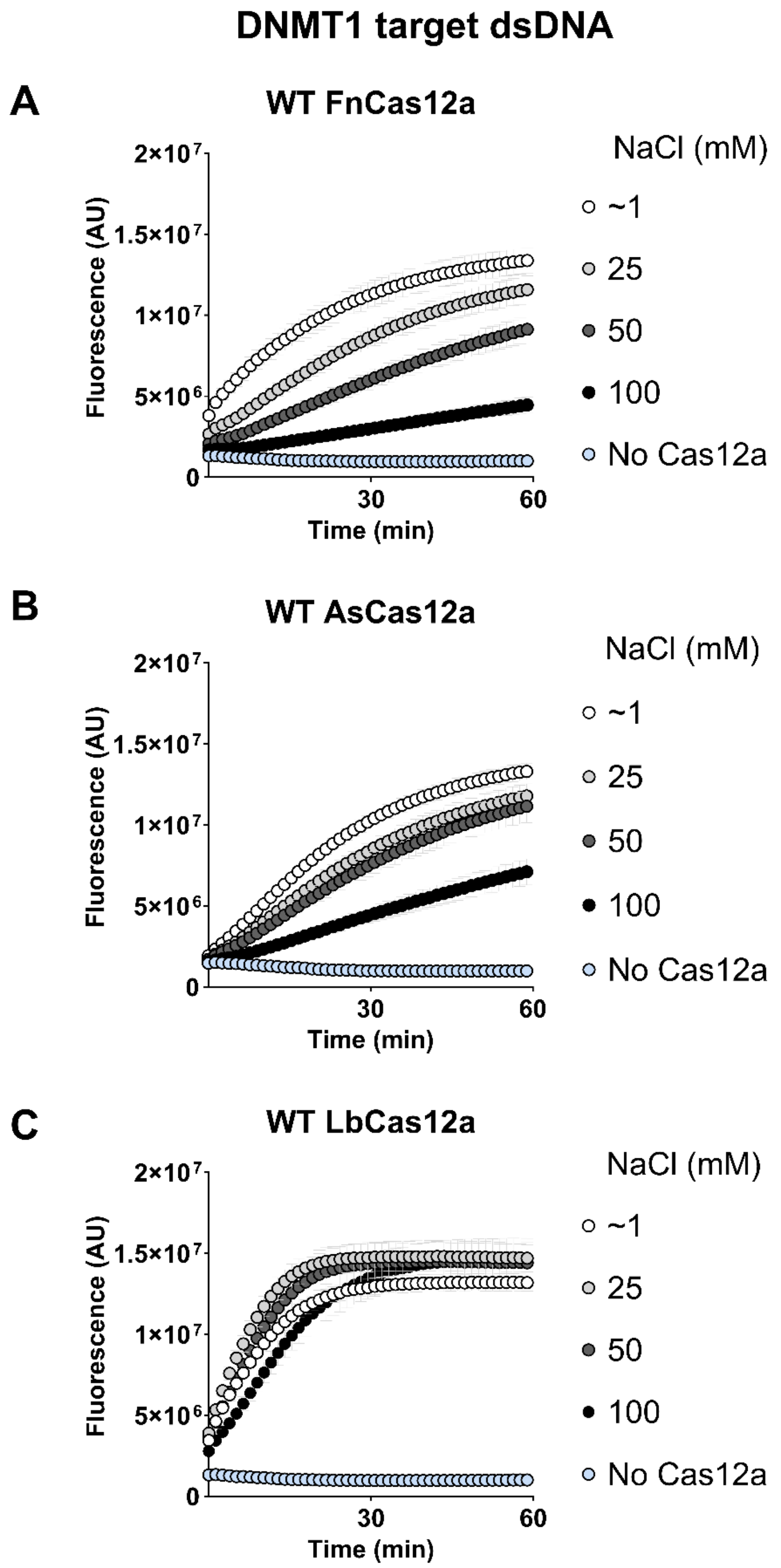

Figure S8: *Trans* cleavage curves of (**A**) WT FnCas12a, (**B**) WT AsCas12a, and (**C**) WT LbCas12a when activated by 1 nM DNMT1 dsDNA, in reaction buffers containing a final NaCl concentration of ~1 to 100 mM NaCl.

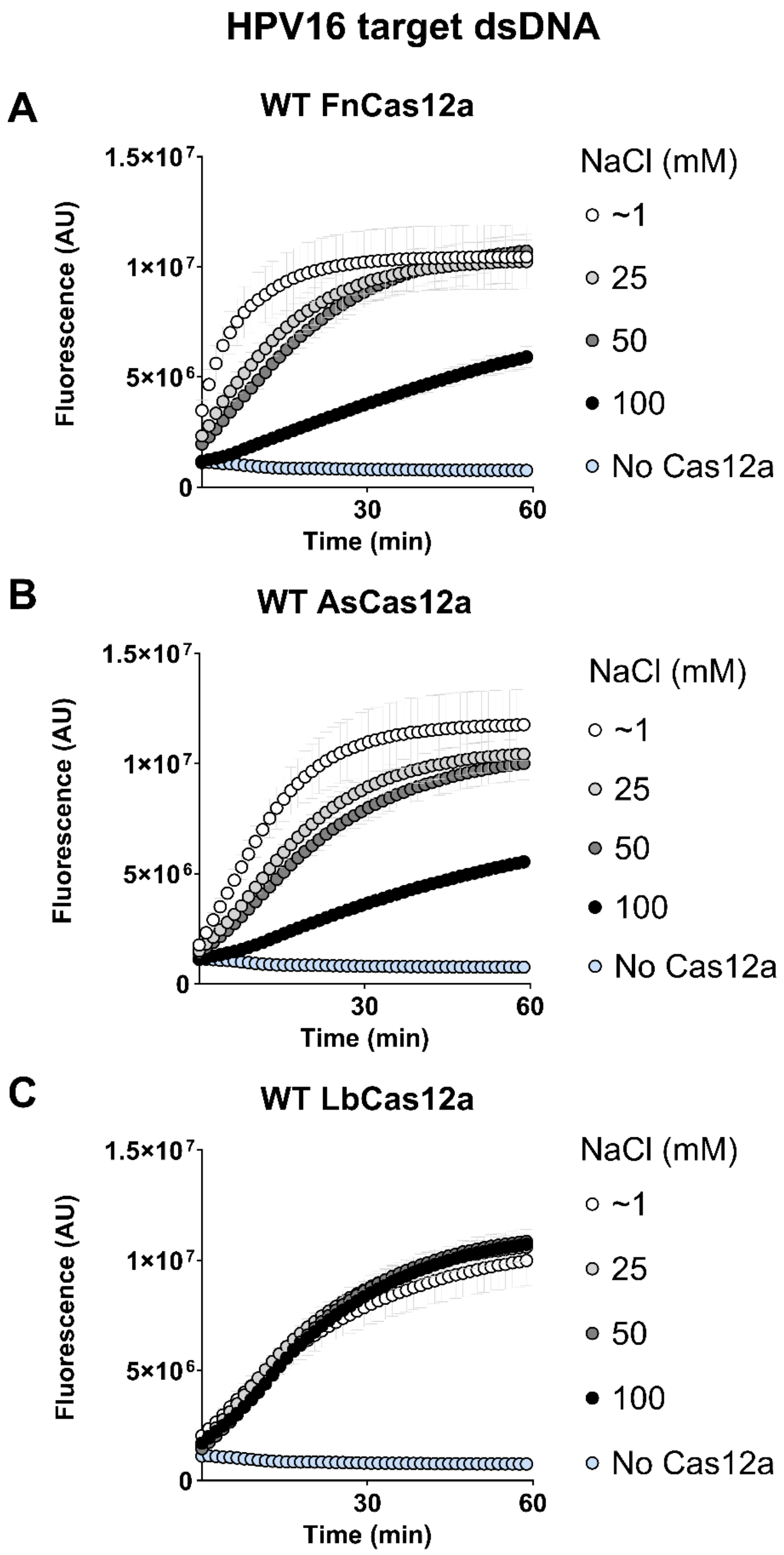

Figure S9: *Trans* cleavage curves of (**A**) WT FnCas12a, (**B**) WT AsCas12a, and (**C**) WT LbCas12a when activated by 1 nM HPV16 dsDNA, in reaction buffers containing a final NaCl concentration of ~1 to 100 mM NaCl.

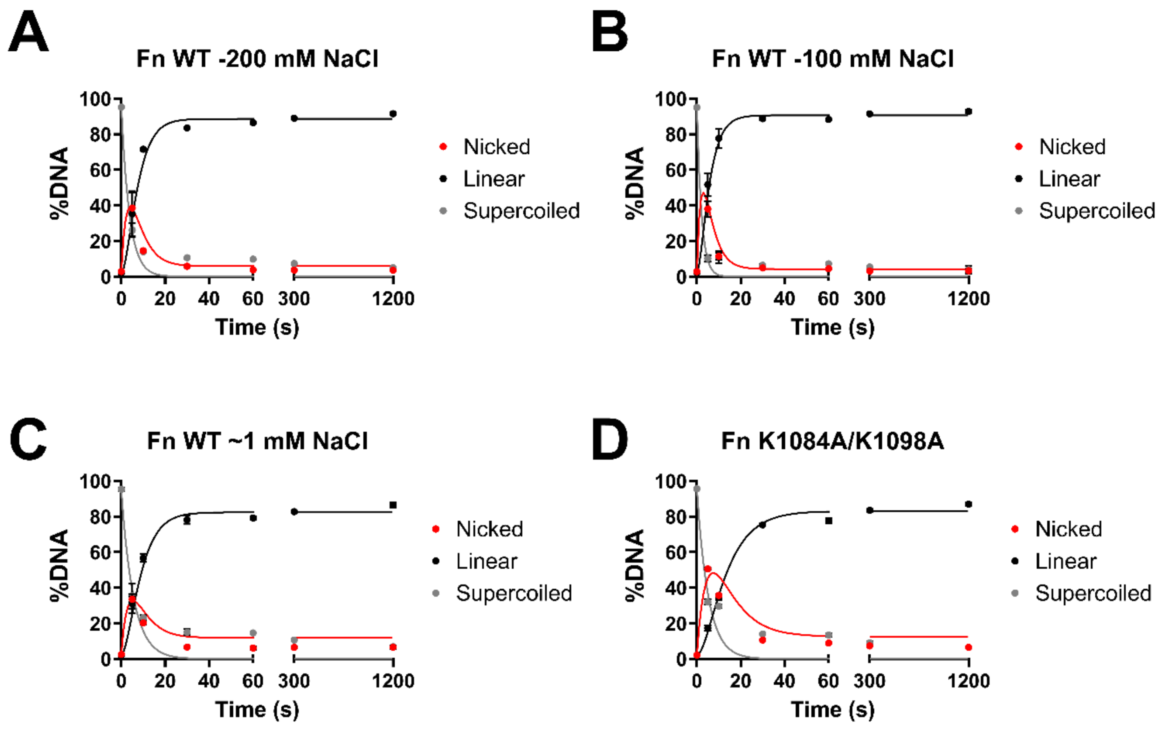

Figure S10: Time-course plasmid *cis* cleavage of WT FnCas12a, in reaction buffers containing a final NaCl concentration of (**A**) 200 mM, (**B**) 100 mM, (**C**) ~1 mM. (**D**) *Cis* cleavage kinetics of FnCas12a K1084A/K1098A mutant, in a final concentration of 50 mM NaCl. Dots show mean, error bars show s.d. Lines show fit of sequential NTS and TS cleavage model.

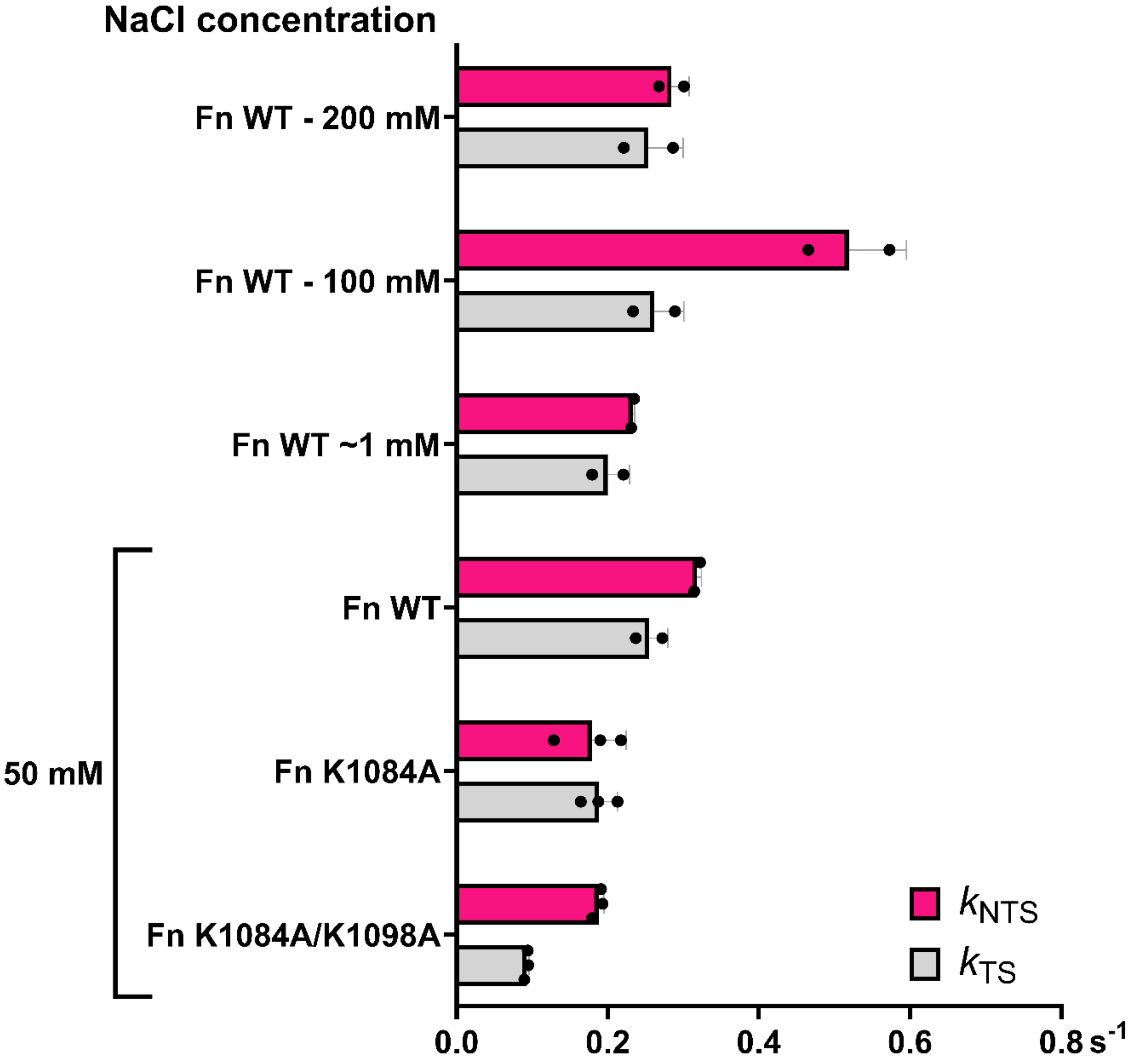

Figure S11: Kinetic values (s^{-1}) of NTS (k_{NTS}, pink) and TS cleavage (k_{TS}, grey), for the Cas12a indicated. Dots show individual replicates, bars show mean, error bars show s.d. Derived from **Figure S6A** and **Figure S10** for the sample indicated.

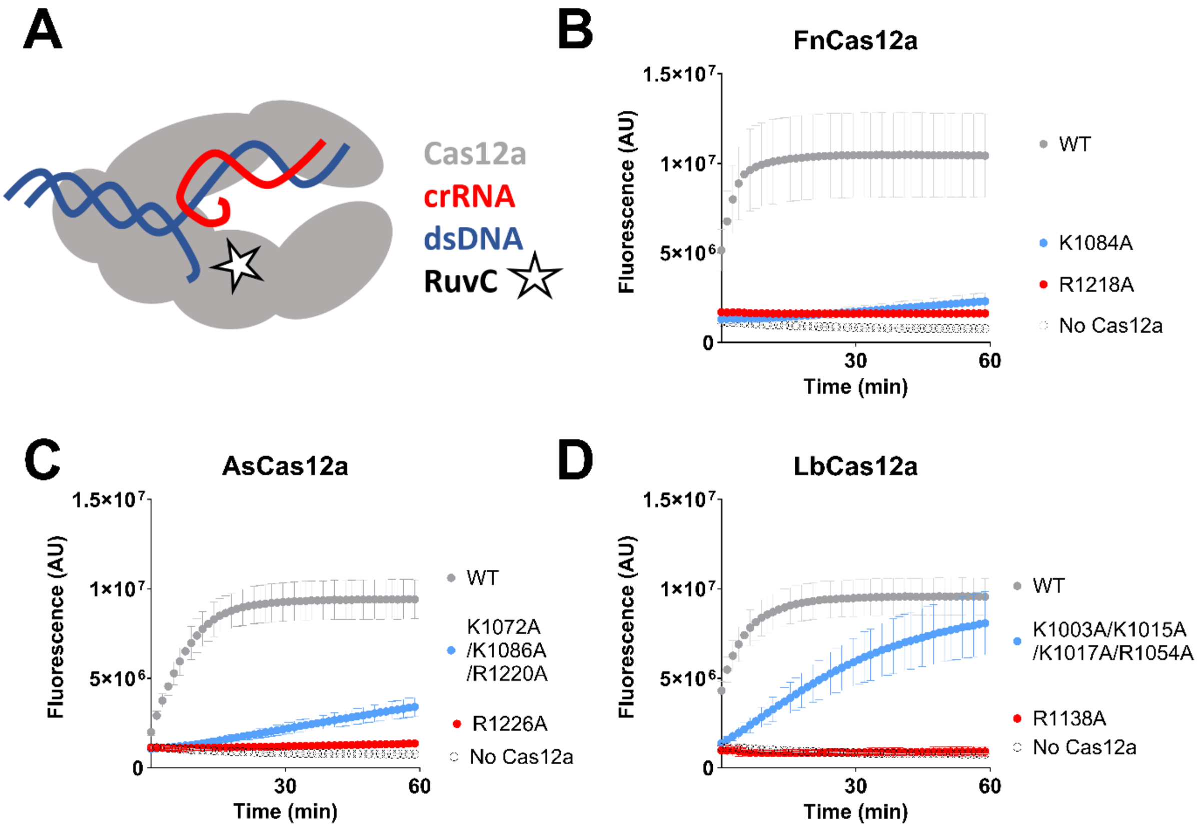

Figure S12: (**A**) Diagram of Cas12a bound to a 'pre-cleaved' dsDNA substrate. *Trans* cleavage of (**B**) FnCas12a WT and mutants, (**C**) AsCas12a WT and mutants, (**D**) LbCas12a WT and mutants, when activated by 1 nM 'pre-cleaved' dsDNA substrate.

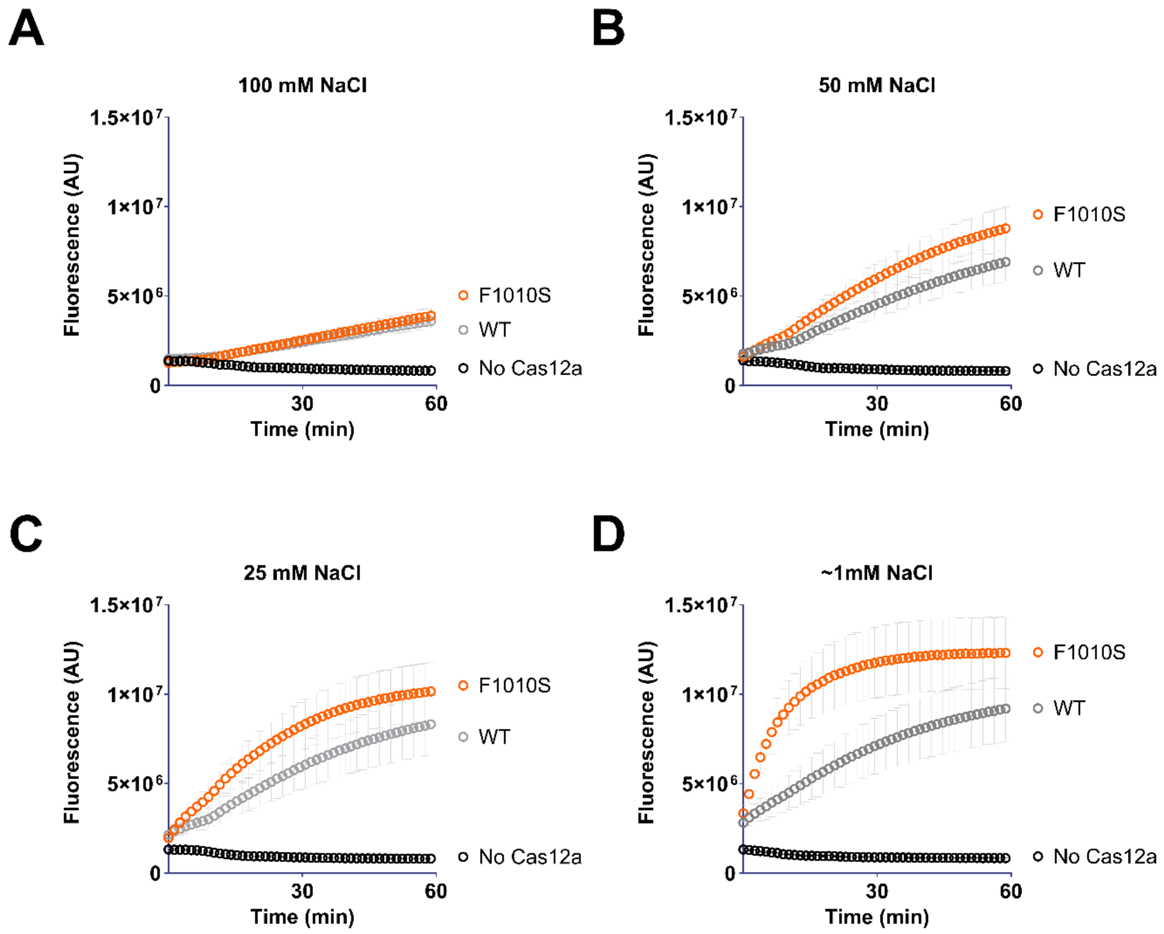

Figure S13: *Trans* cleavage curves of WT FnCas12a and F1010S mutant, when activated by 1 nM DNMT1 dsDNA, in reaction buffers containing a final NaCl concentration of (**A**) 100 mM, (**B**) 50 mM, (**C**) 25 mM, (**D**) ~1 mM.

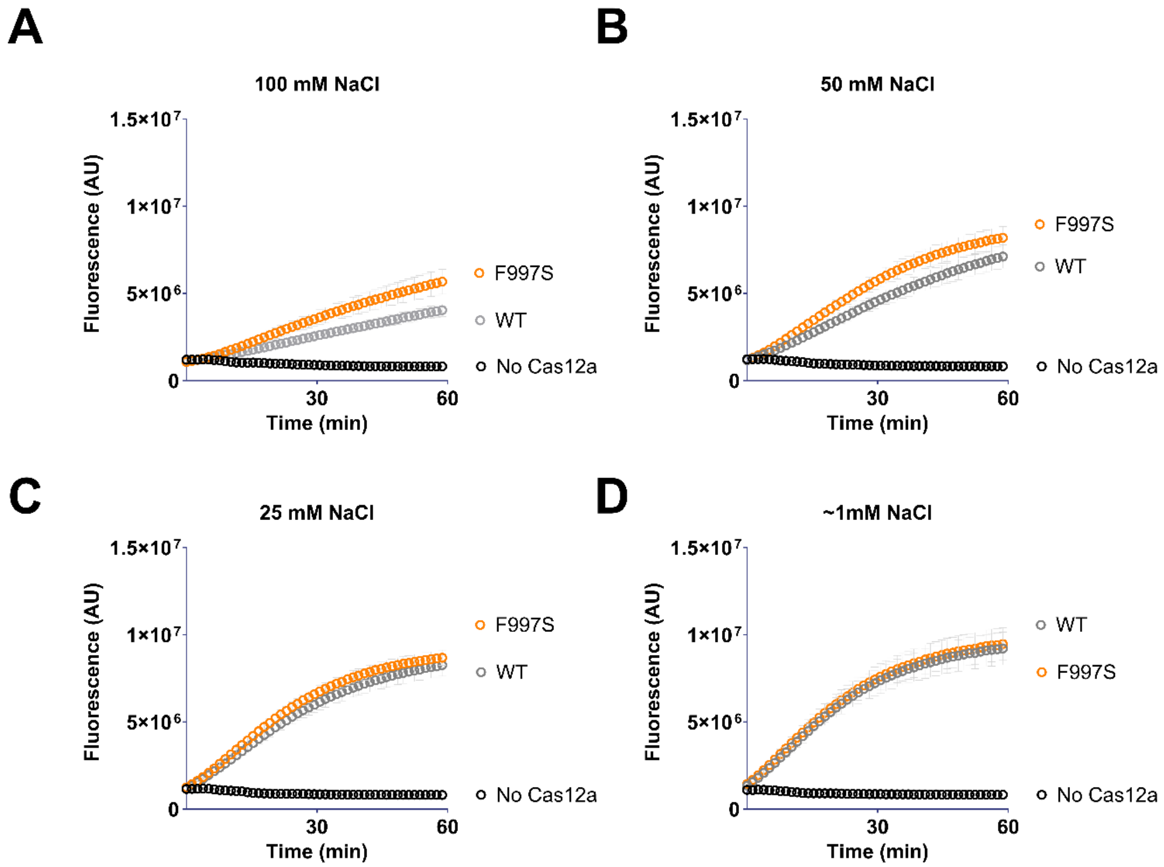

Figure S14: *Trans* cleavage curves of WT AsCas12a and F997S mutant, when activated by 1 nM DNMT1 dsDNA, in reaction buffers containing a final NaCl concentration of (**A**) 100 mM, (**B**) 50 mM, (**C**) 25 mM, (**D**) ~1 mM.

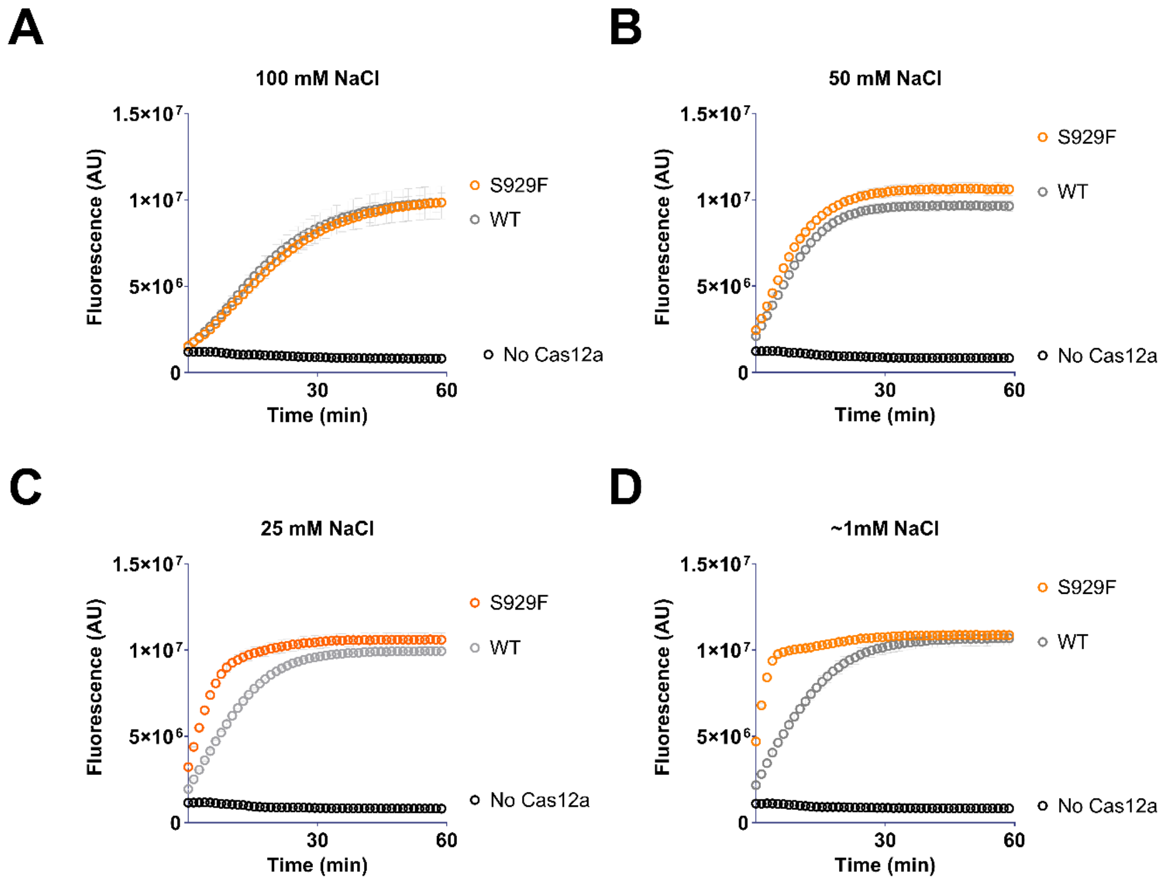

Figure S15: *Trans* cleavage curves of WT LbCas12a and S929F mutant, when activated by 1 nM DNMT1 dsDNA, in reaction buffers containing a final NaCl concentration of (**A**) 100 mM, (**B**) 50 mM, (**C**) 25 mM, (**D**) ~1 mM.

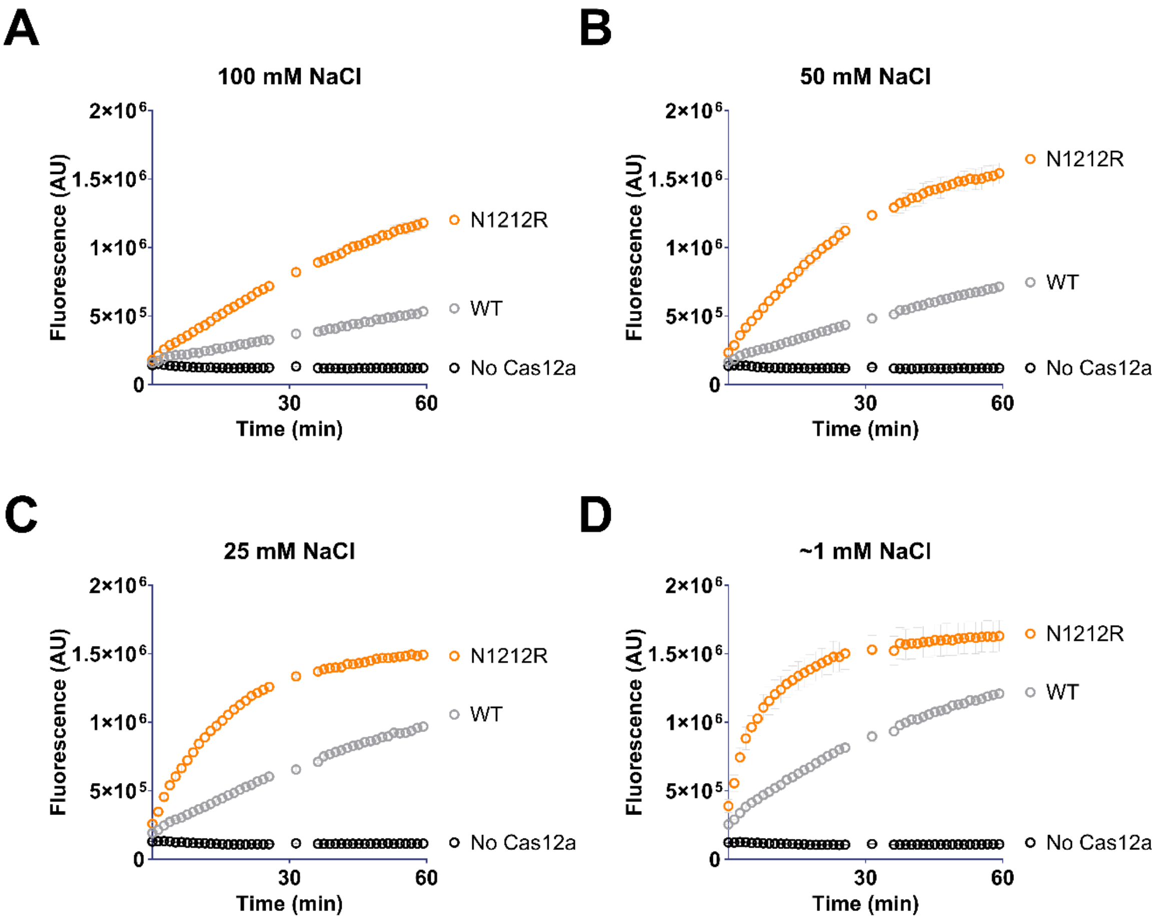

Figure S16: *Trans* cleavage curves of WT FnCas12a and N1212R mutant, when activated by 1 nM DNMT1 dsDNA, in reaction buffers containing a final NaCl concentration of (**A**) 100 mM, (**B**) 50 mM, (**C**) 25 mM, (**D**) ~1 mM. Missing data points due to machine error.

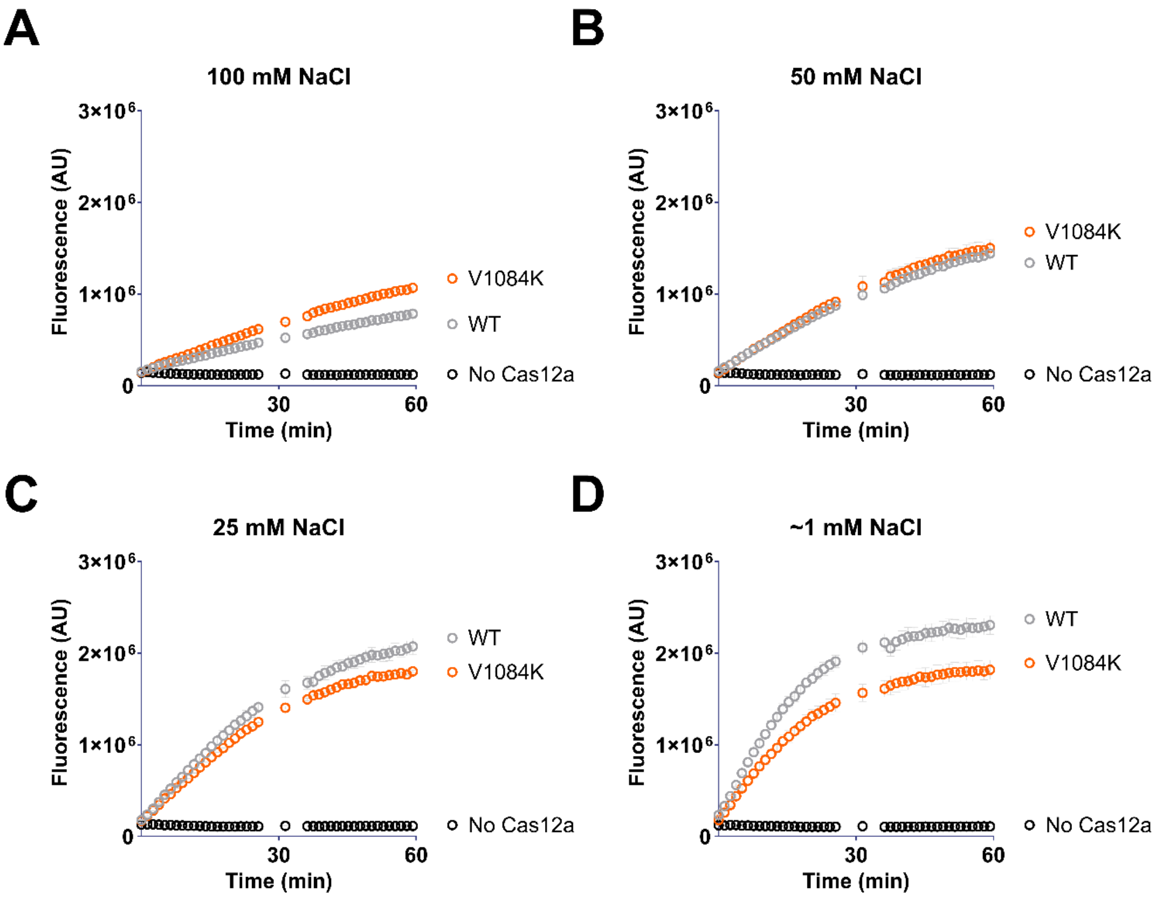

Figure S17: *Trans* cleavage curves of WT AsCas12a and V1084K mutant, when activated by 1 nM DNMT1 dsDNA, in reaction buffers containing a final NaCl concentration of (**A**) 100 mM, (**B**) 50 mM, (**C**) 25 mM, (**D**) ~1 mM. Missing data points due to machine error.

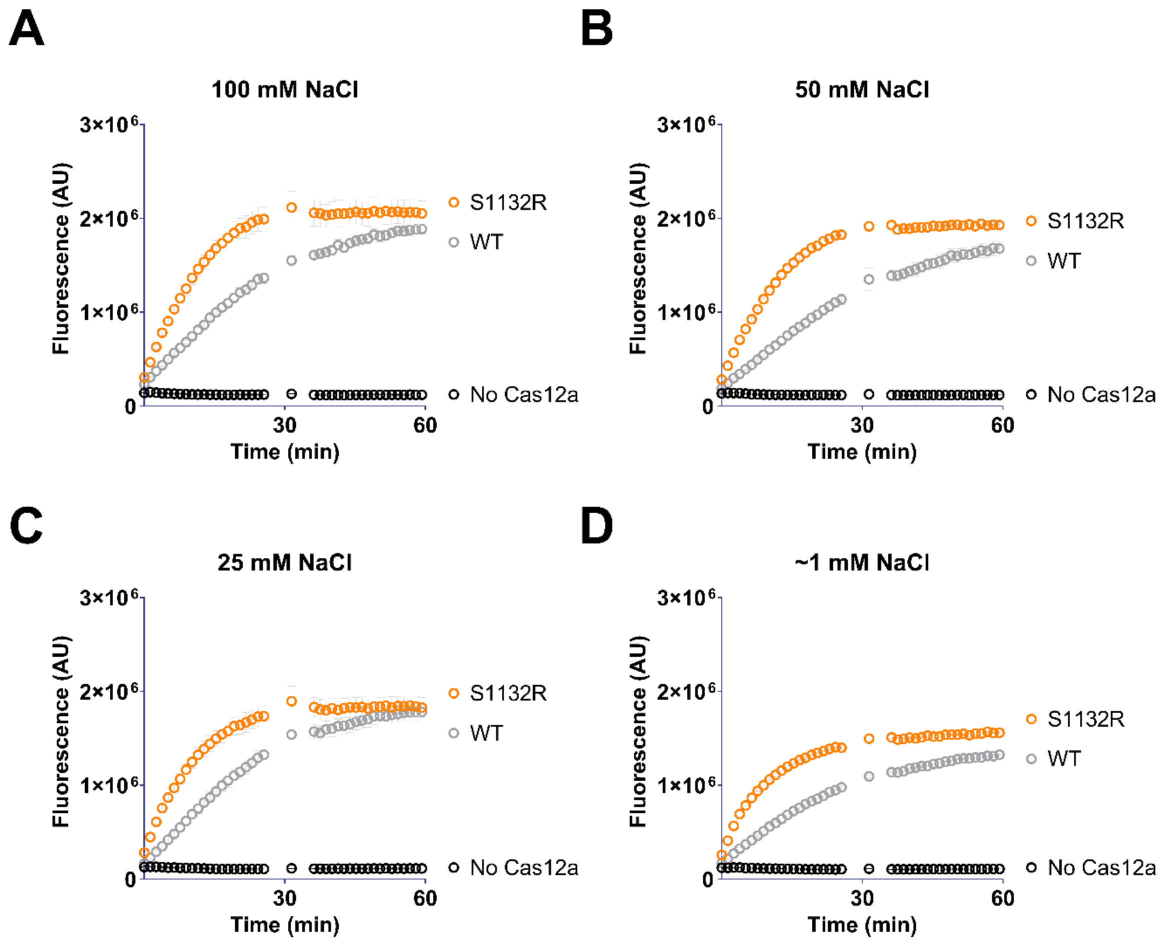

Figure S18: *Trans* cleavage curves of WT LbCas12a and S1132R mutant, when activated by 1 nM DNMT1 dsDNA, in reaction buffers containing a final NaCl concentration of (**A**) 100 mM, (**B**) 50 mM, (**C**) 25 mM, (**D**) ~1 mM. Missing data points due to machine error.

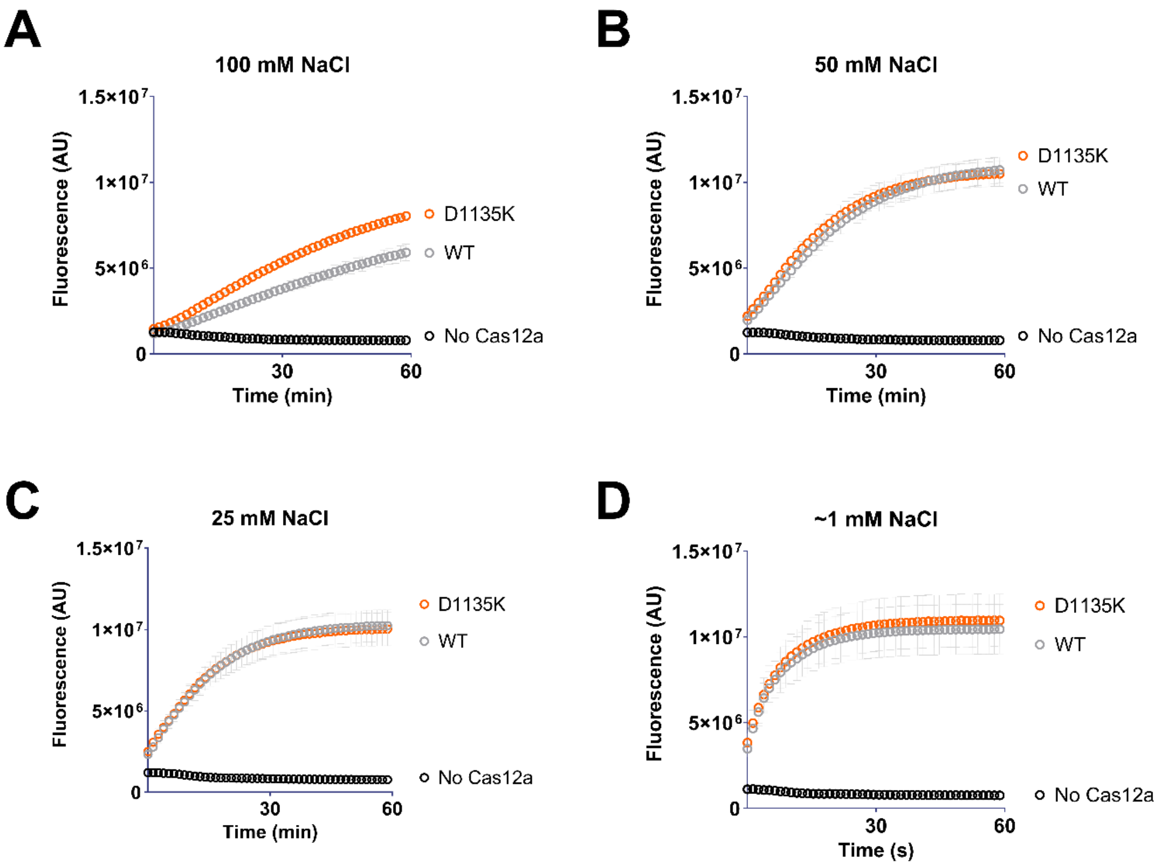

Figure S19: *Trans* cleavage curves of WT FnCas12a, and D1135K mutant, when activated by 1 nM DNMT1 dsDNA, in reaction buffers containing a final NaCl concentration of (**A**) 100 mM, (**B**) 50 mM, (**C**) 25 mM, (**D**) ~1 mM.

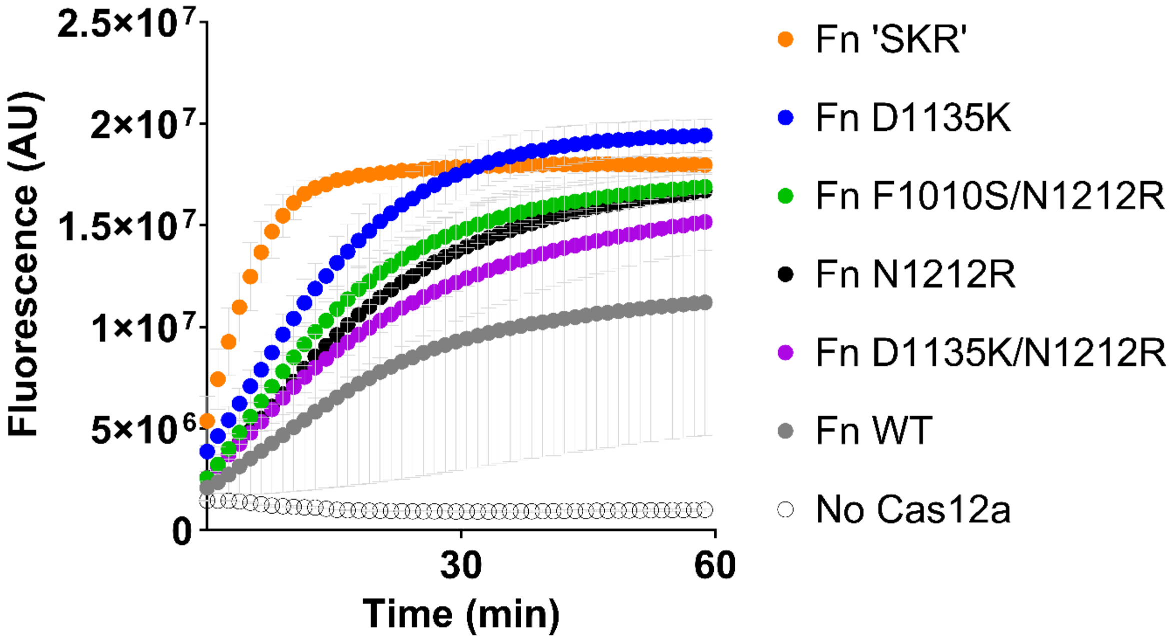

Figure S20: *Trans* cleavage curves of FnCas12a WT and mutants (as indicated), when activated by 1 nM DNMT1 dsDNA, in reaction buffer containing 50 mM NaCl.

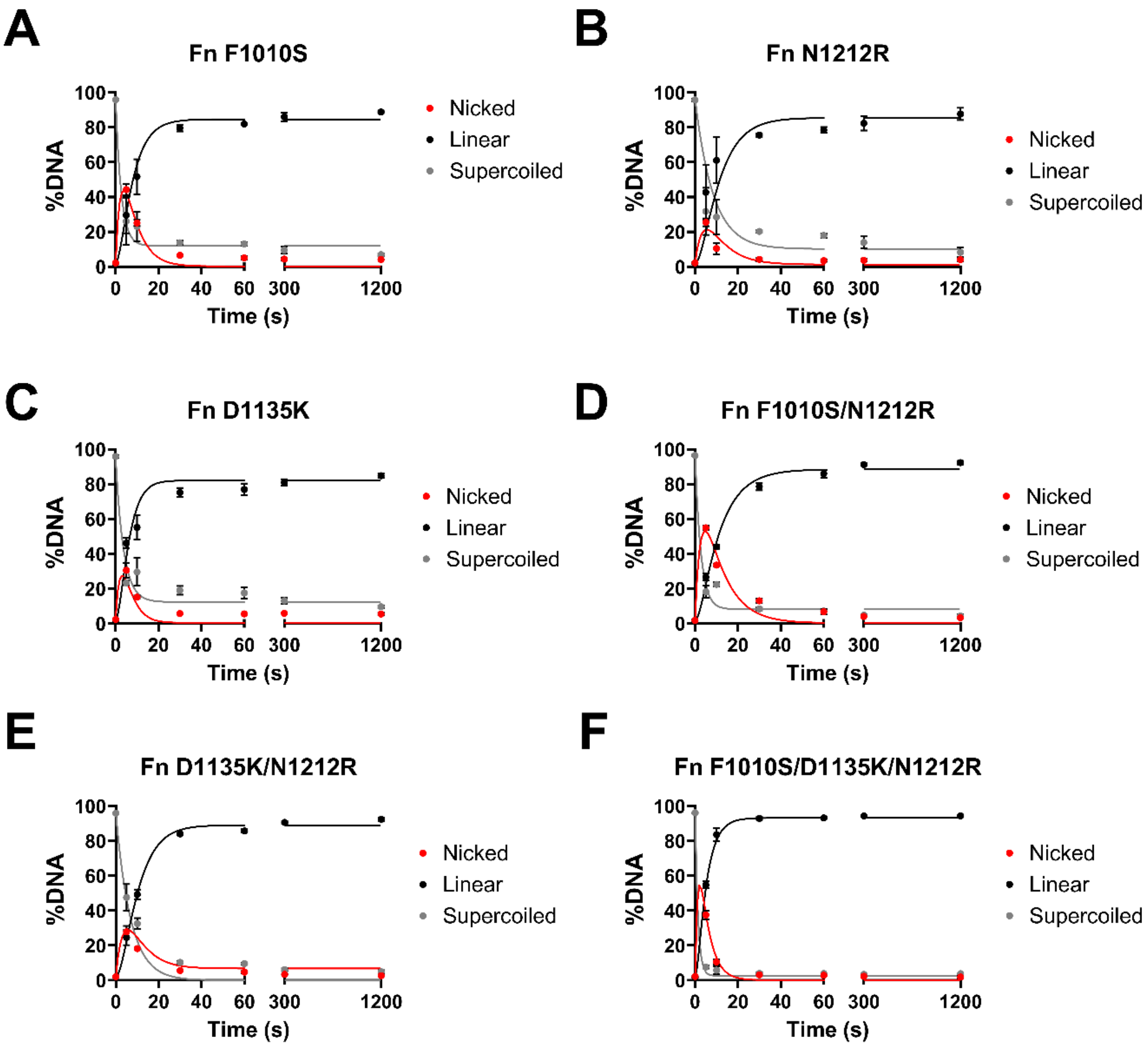

Figure S21: Time-course plasmid *cis* cleavage by (**A**) FnF1010S, (**B**) FnN1212R, (**C**) FnD1135K, (**D**) FnF1010S/N1212R, (**E**) FnD1135K/N1212R, and (**F**) FnF1010S/D1135K/N1212R. Dots show mean, error bars show s.d. Lines show fit of sequential NTS and TS cleavage model. Conducted in reaction buffers with a final concentration of 50 mM NaCl.

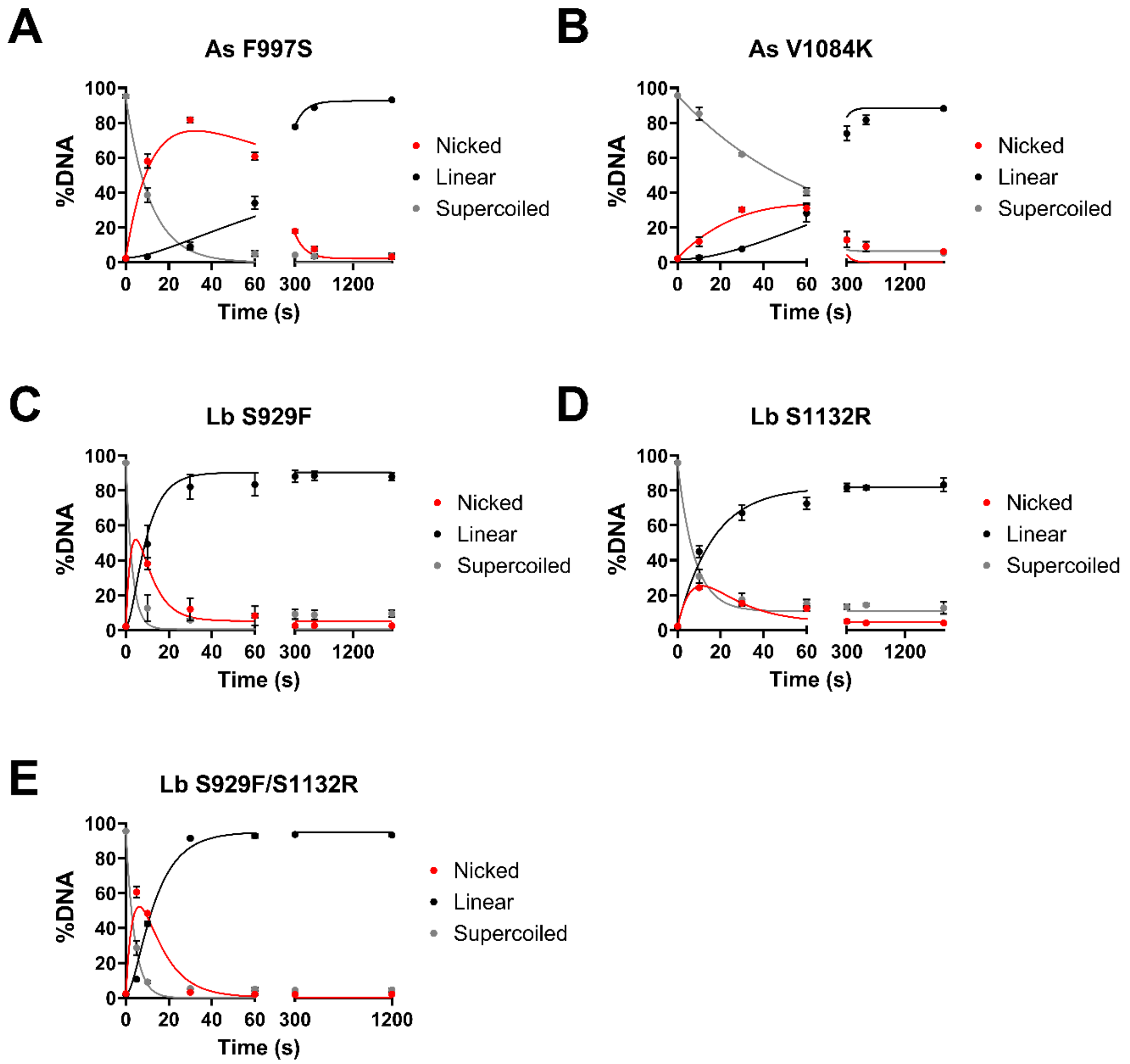

Figure S22: Time-course plasmid *cis* cleavage by (**A**) AsF997S, (**B**) AsV1084K, (**C**) LbS929F, (**D**) LbS1132R, (**E**) LbS929F/S1132R. Dots show mean, error bars show s.d. Lines show fit of sequential NTS and TS cleavage model. Conducted in reaction buffers with a final concentration of 50 mM NaCl.

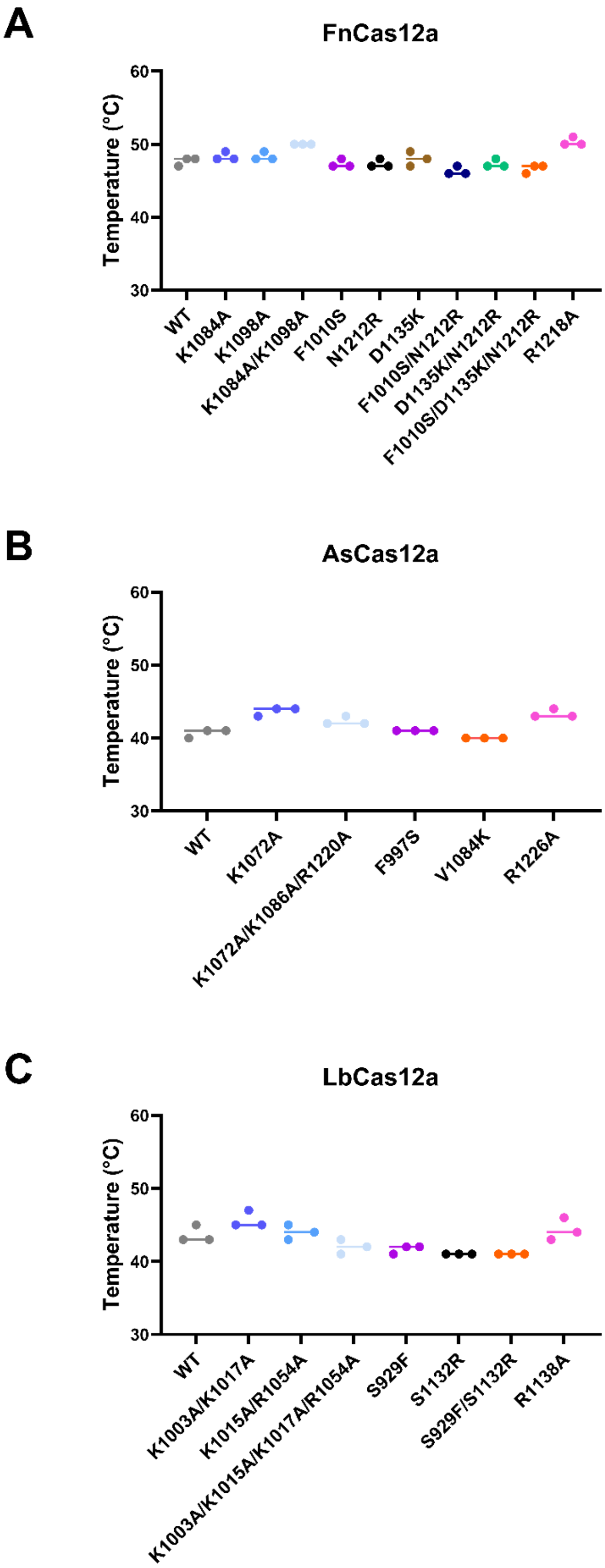

Figure S23: Protein thermostability, where melting temperature (°C) is defined as the fluorescence peak. Line shows mean of three replicates.

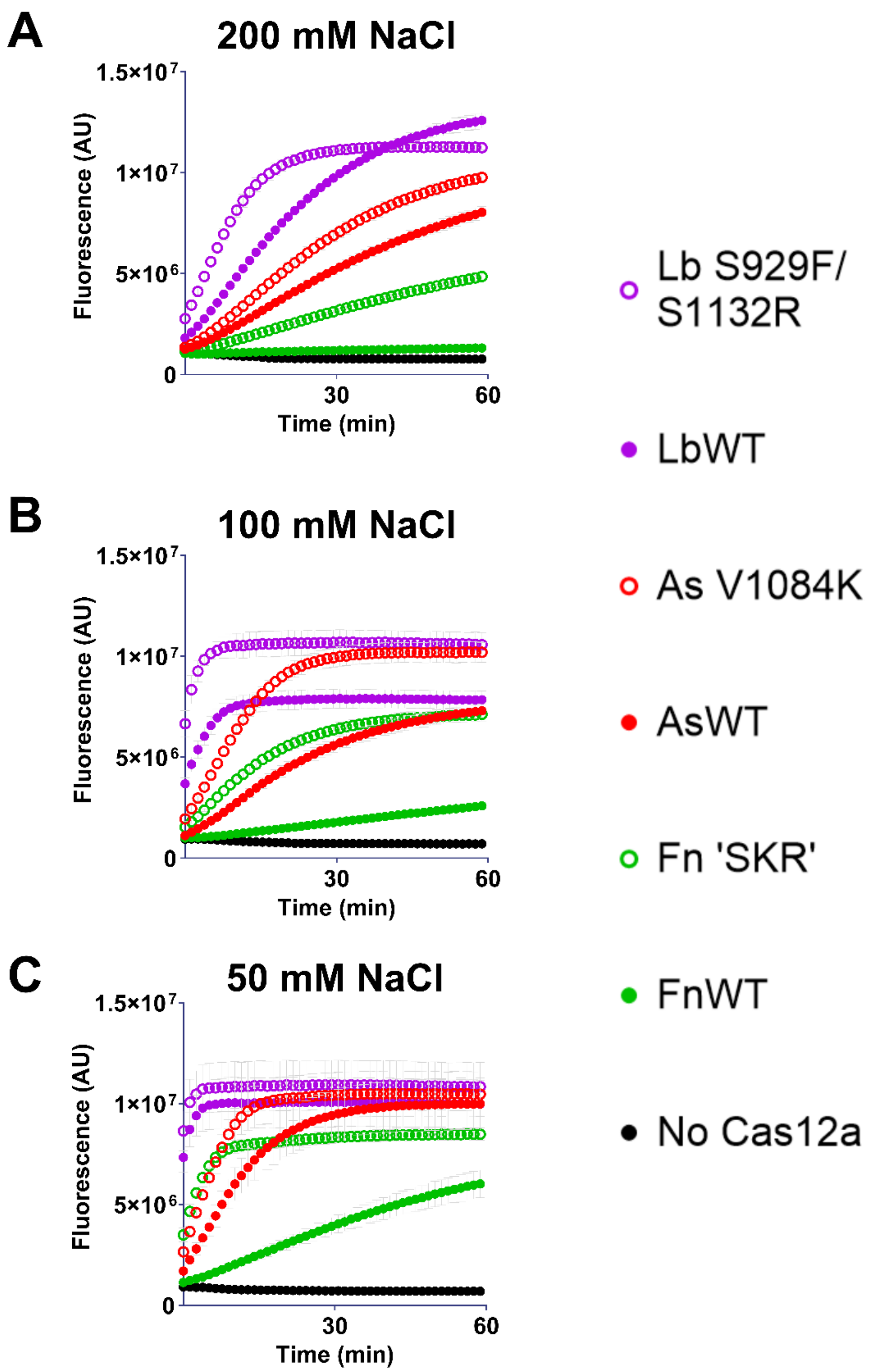

Figure S24: *Trans* cleavage curves of Cas12a WT and mutants (as indicated), when activated by 1 nM HPV16 dsDNA, in reaction buffers containing a final NaCl concentration of (**A**) 200 mM, (**B**) 100 mM, and (**C**) 50 mM.

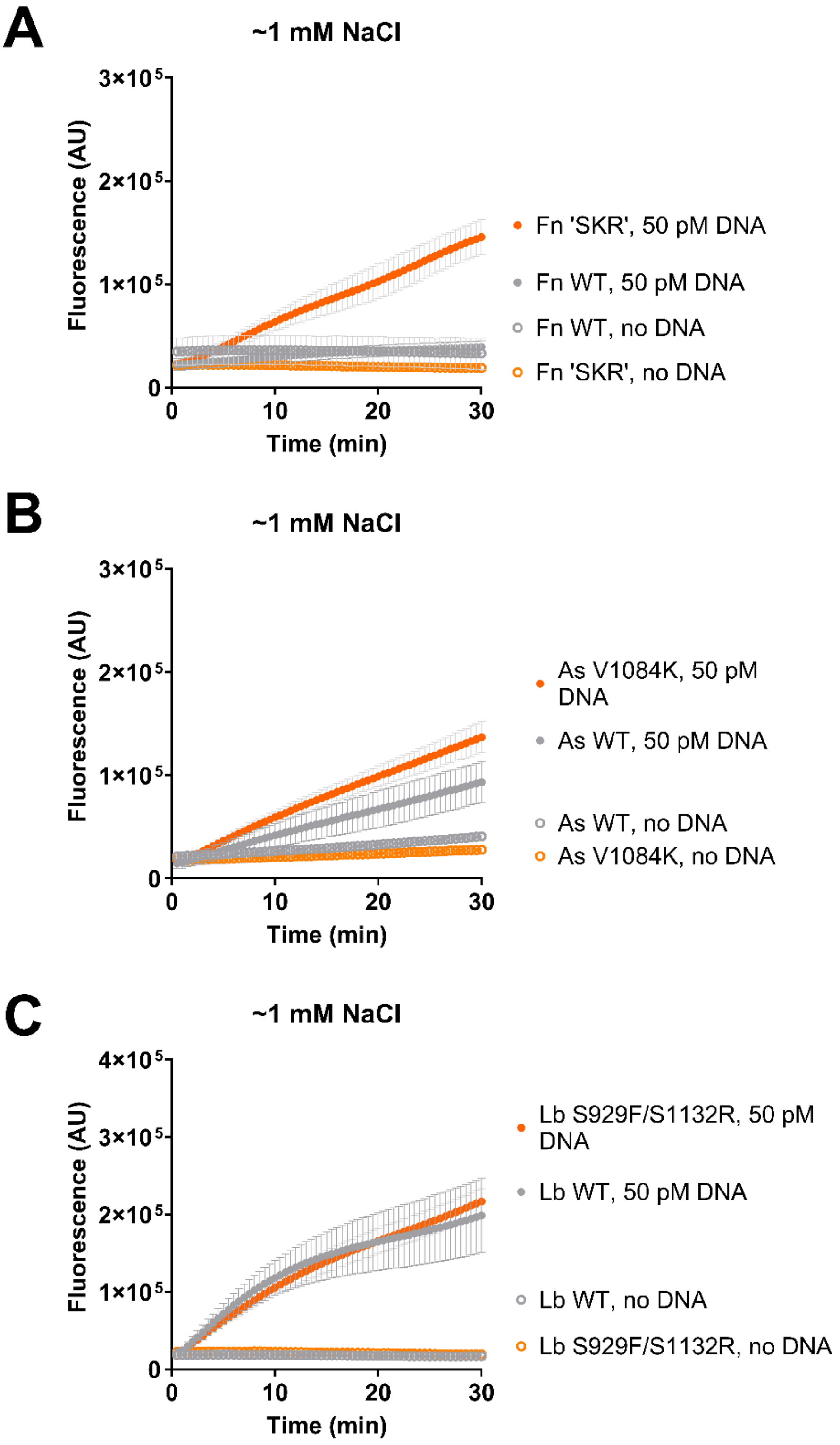

Figure S25: *Trans* cleavage curves in ~1 mM NaCl reaction buffer; for **A**) FnCas12a WT and 'SKR' mutant, (**B**) AsCas12a WT and V1084K mutant, and (**C**) LbCas12a WT and S929F/S1132R mutant, in the presence of either no DNA or 50 pM HPV16 dsDNA (as indicated).

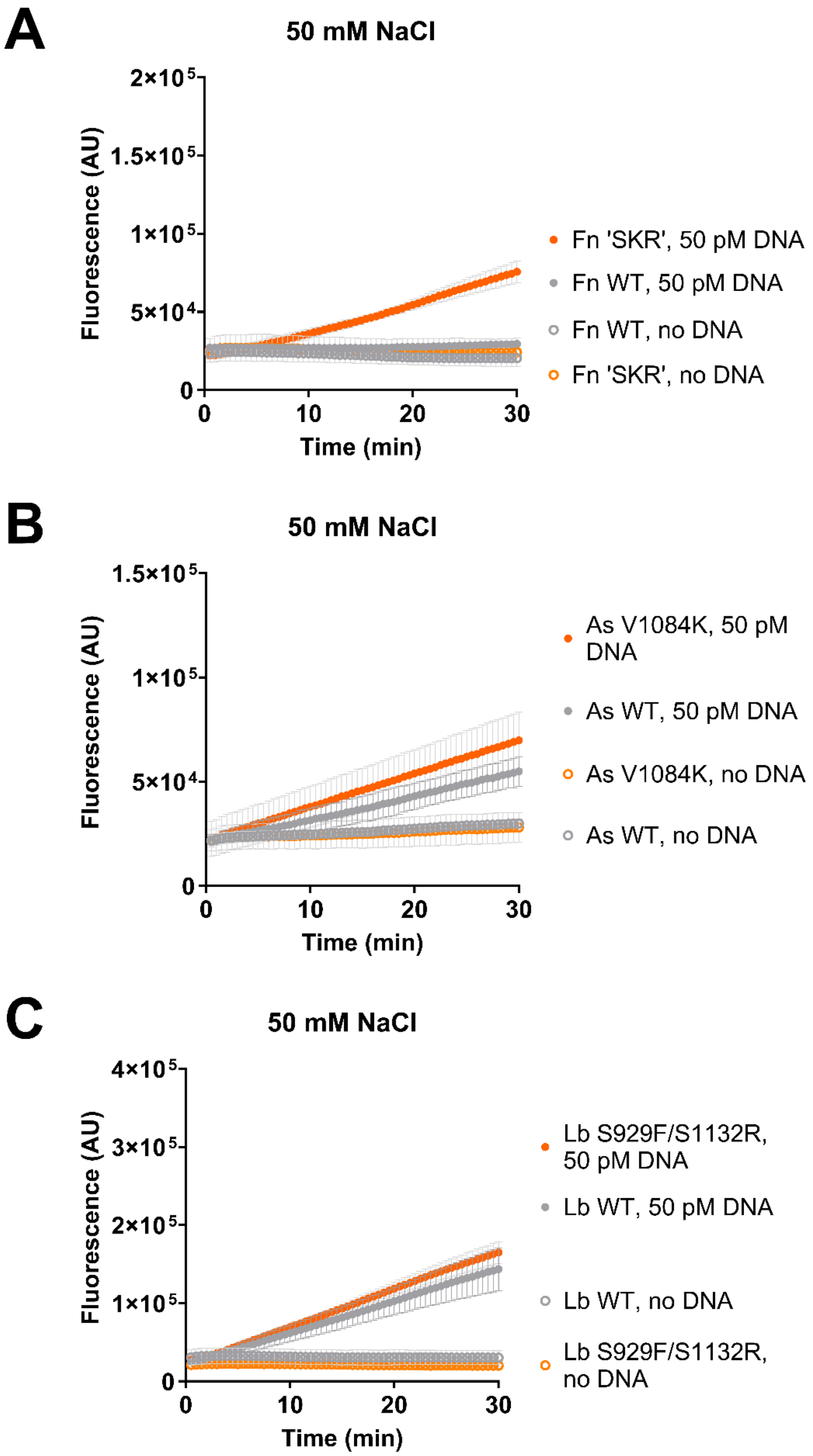

Figure S26: *Trans* cleavage curves in 50 mM NaCl reaction buffer; for **A**) FnCas12a WT and 'SKR' mutant, (**B**) AsCas12a WT and V1084K mutant, and (**C**) LbCas12a WT and S929F/S1132R mutant, in the presence of either no DNA or 50 pM HPV16 dsDNA (as indicated).

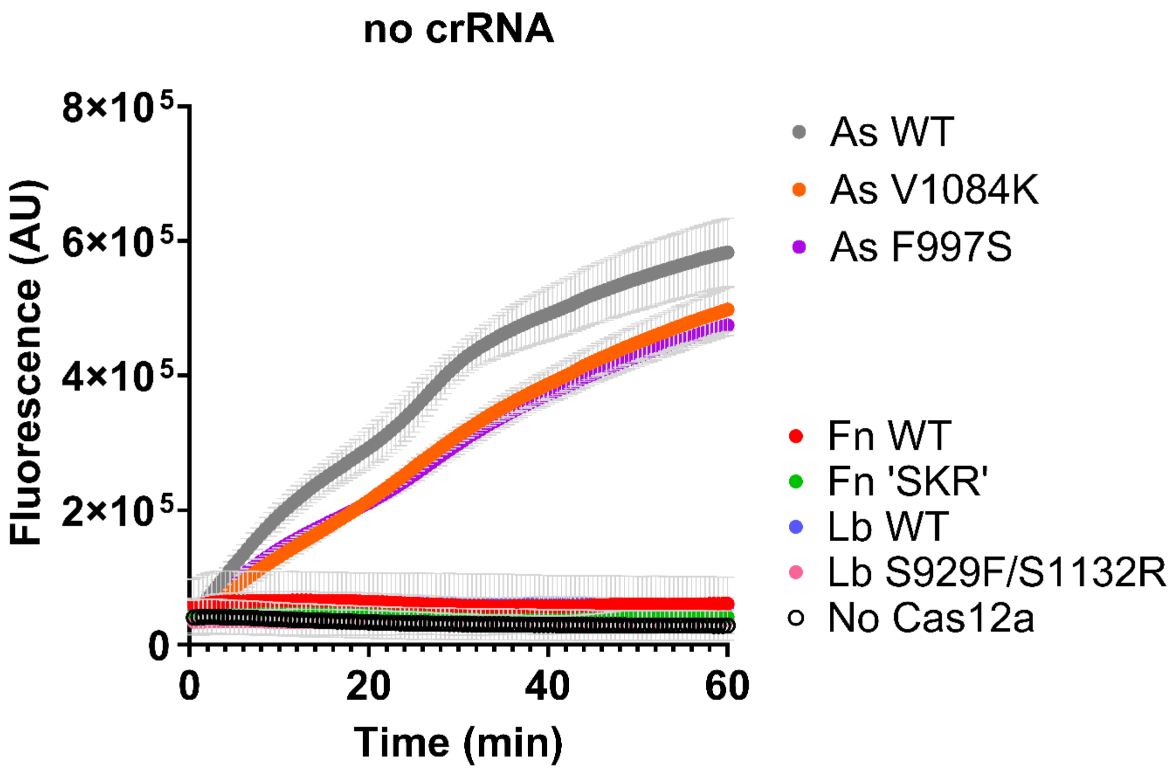

Figure S27: *Trans* cleavage curves of Cas12a WT and mutants (as indicated), in the absence of crRNA or any target dsDNA, reaction buffers containing a final NaCl concentration of ~1 mM.

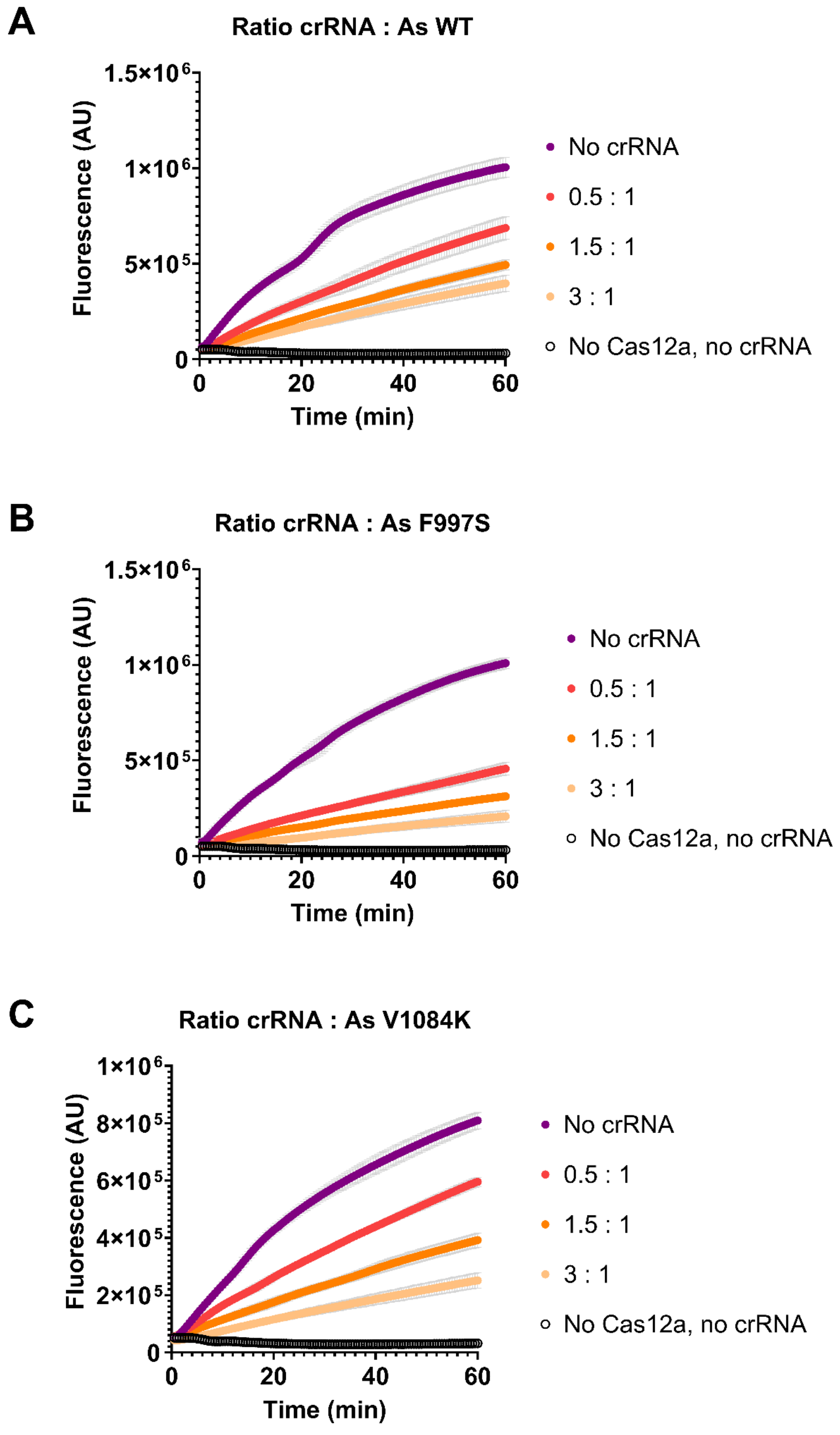

Figure S28: *Trans* cleavage curves of AsCas12a (**A**) WT, (**B**) F997S, and (**C**) V1084K, in the absence of any target dsDNA, and molar ratio of crRNA to Cas12a as indicated.

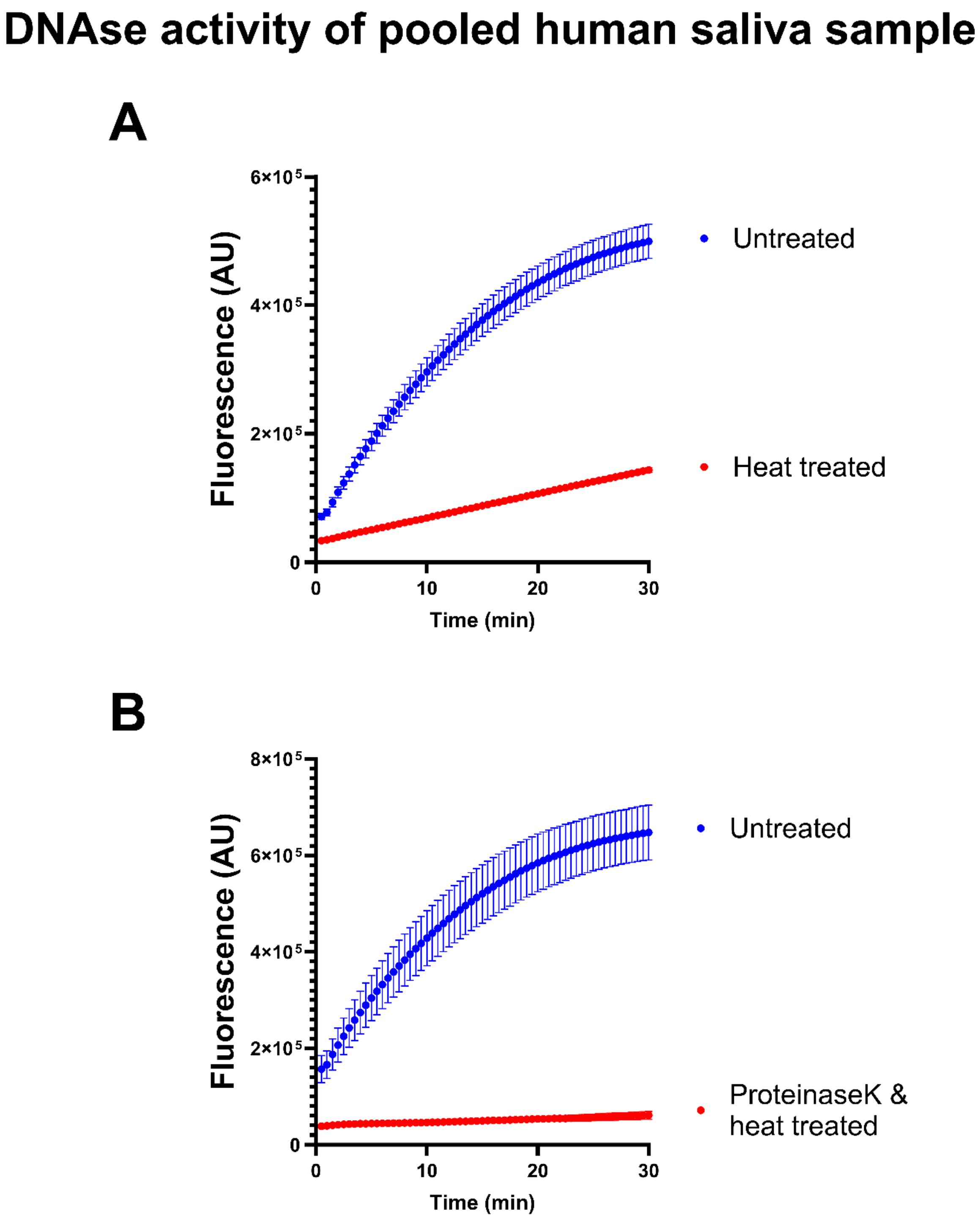

Figure S29: Troubleshooting the DNAse activity of pooled human saliva sample, comparing (**A**) untreated vs heat-treated at 98 °C for 10 mins, and (**B**) untreated vs addition of 2% v/v proteinase K and incubation at 55 °C for 10 mins, followed by heat-treatment at 98 °C for 10 mins.

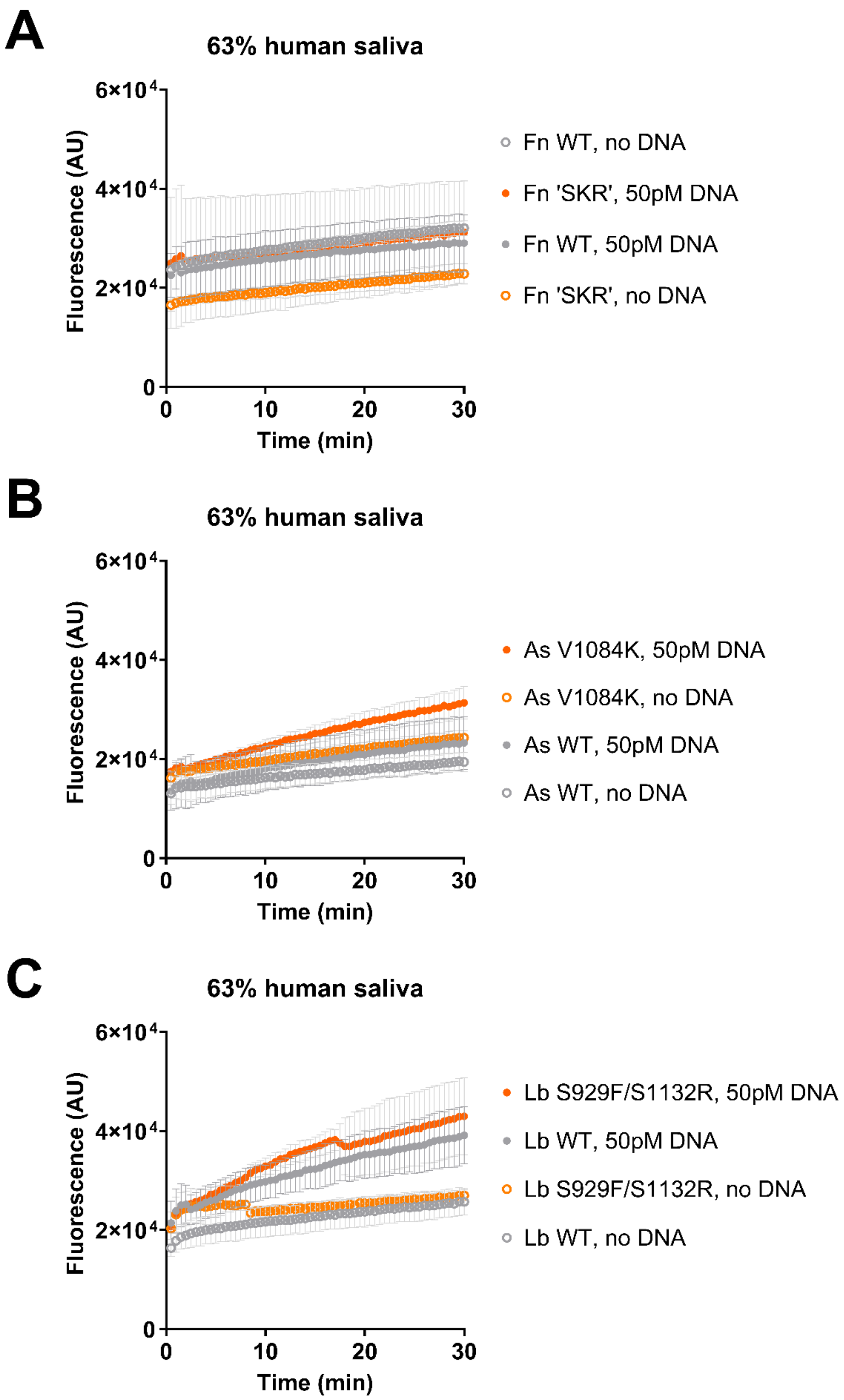

Figure S30: *Trans* cleavage curves in 63% human saliva sample; for **A**) FnCas12a WT and 'SKR' mutant, (**B**) AsCas12a WT and V1084K mutant, and (**C**) LbCas12a WT and S929F/S1132R mutant, spiked with either nuclease-free water or 50 pM HPV16 dsDNA (as indicated).

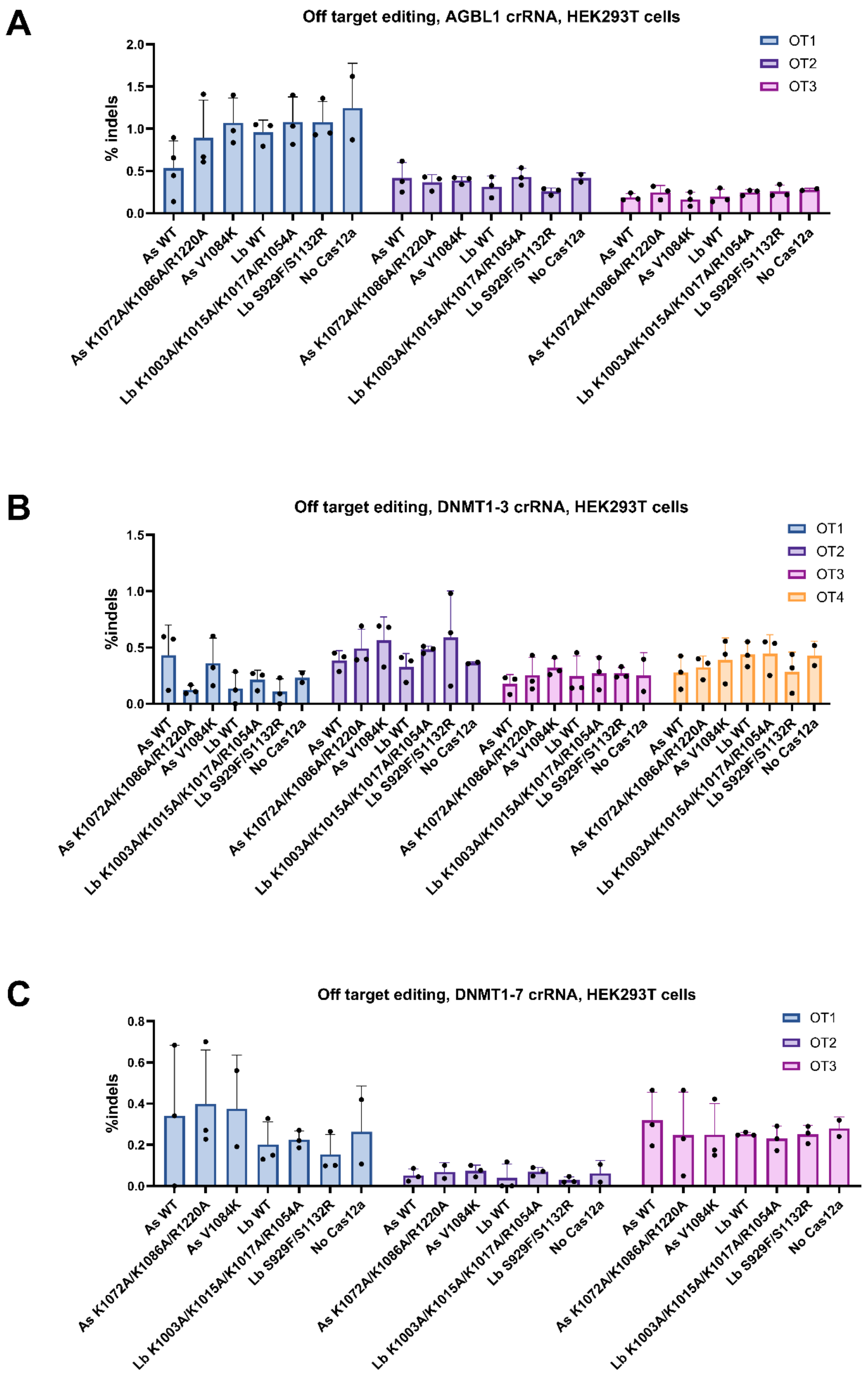

Figure S31: Off-target editing in HEK293T cells. Highest represented off-target (OT) sites for (**A**) AGBL1, (**B**) DNMT1-3, and (**C**) DNMT1-7, as identified in Kleinstiver 2019 (https://doi.org/10.1038/s41587-018-0011-0). Bars shown mean, error bars s.d. Statistical significance evaluated by two-way ANOVA with Tukey's multiple comparison test, p-values shown for $p<0.05$.

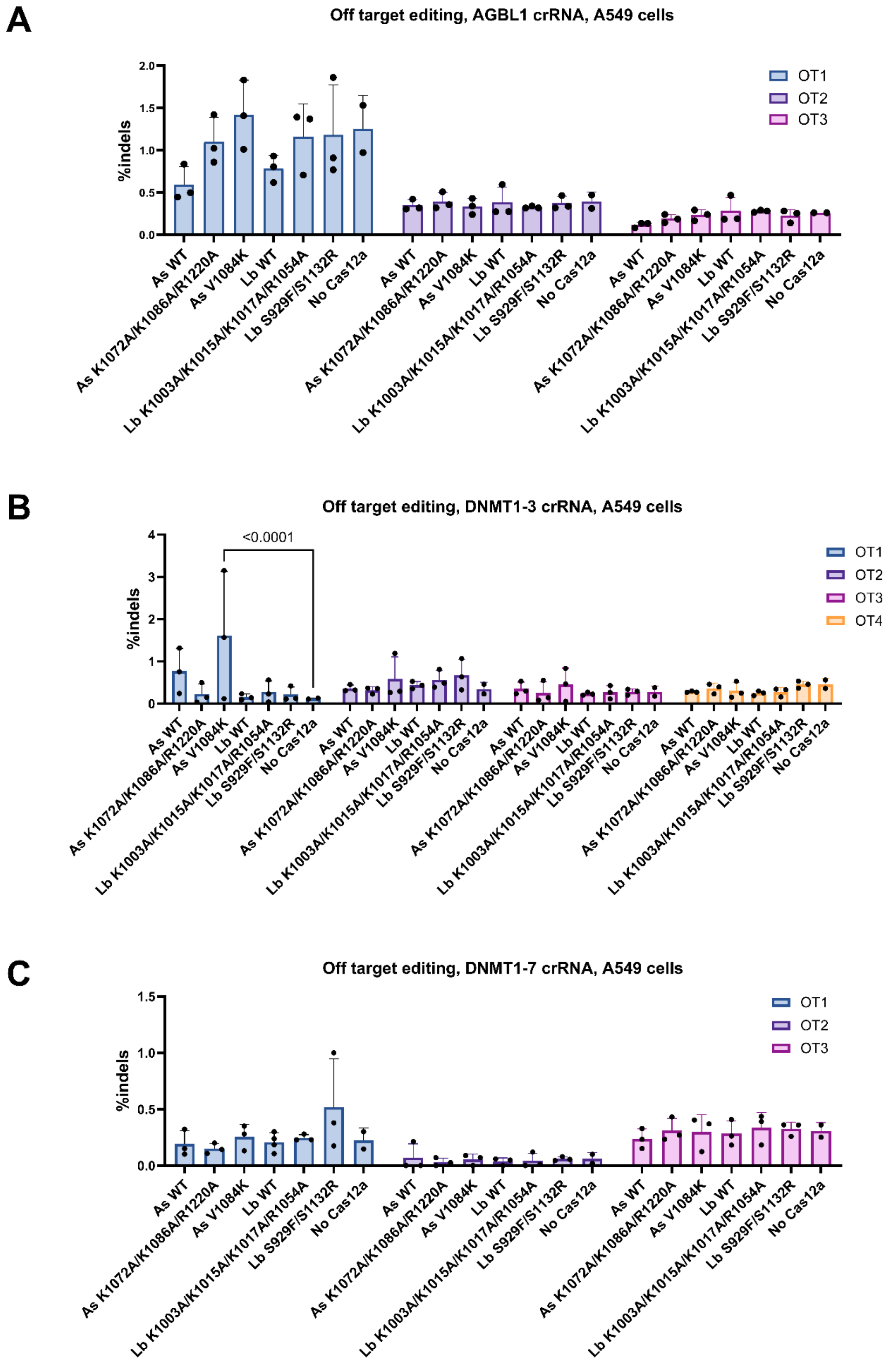

Figure S32: Off-target editing in A549 cells. Highest represented off-target (OT) sites for (**A**) AGBL1, (**B**) DNMT1-3, and (**C**) DNMT1-7, as identified in Kleinstiver 2019 (https://doi.org/10.1038/s41587-018-0011-0). Bars shown mean, error bars s.d. Statistical significance evaluated by two-way ANOVA with Tukey's multiple comparison test, p-values shown for $p<0.05$.

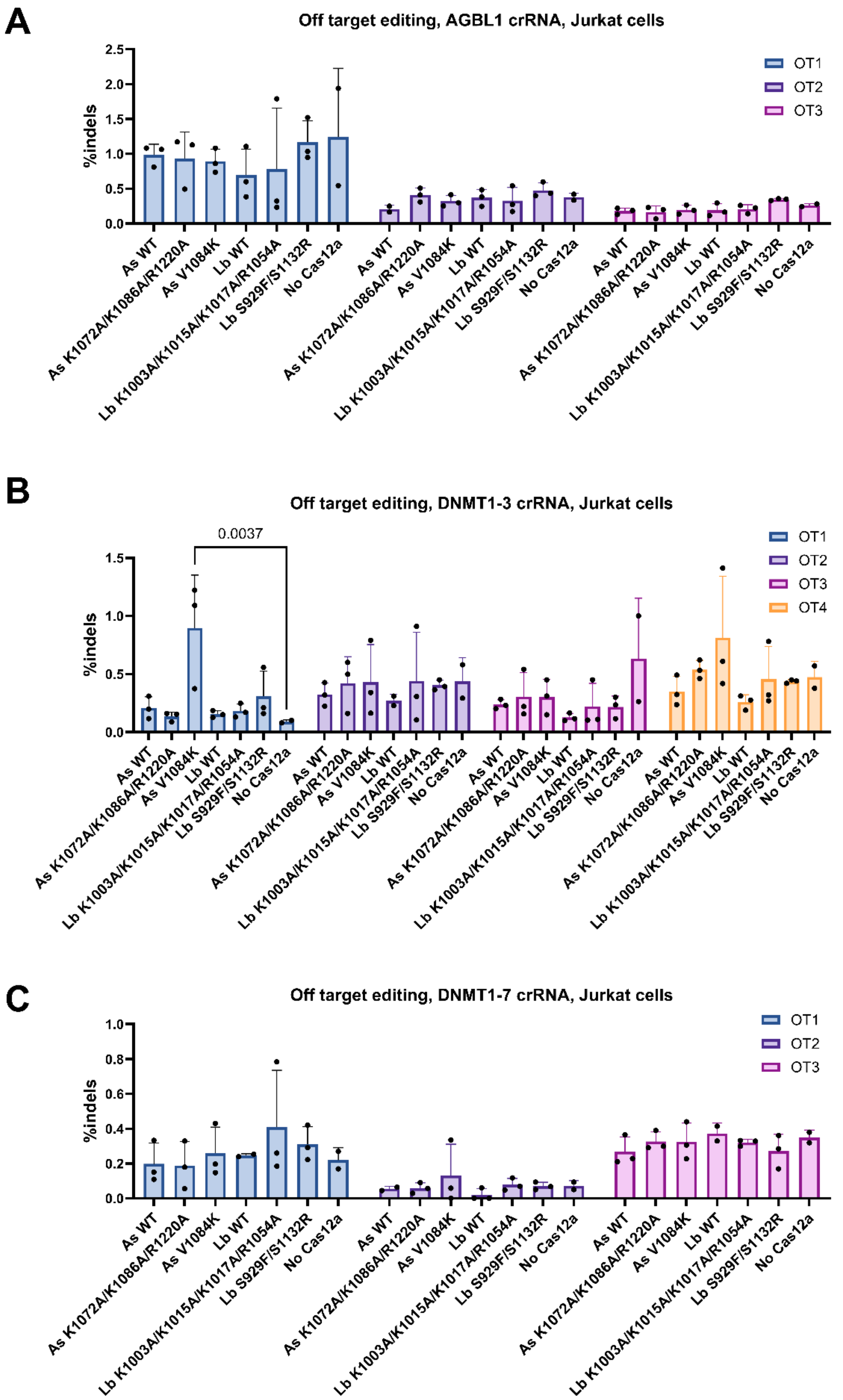

Figure S33: Off-target editing in Jurkat cells. Highest represented off-target (OT) sites for (**A**) AGBL1, (**B**) DNMT1-3, and (**C**) DNMT1-7, as identified in Kleinstiver 2019 (https://doi.org/10.1038/s41587-018-0011-0). Bars shown mean, error bars s.d. Statistical significance evaluated by two-way ANOVA with Tukey's multiple comparison test, p-values shown for $p<0.05$.

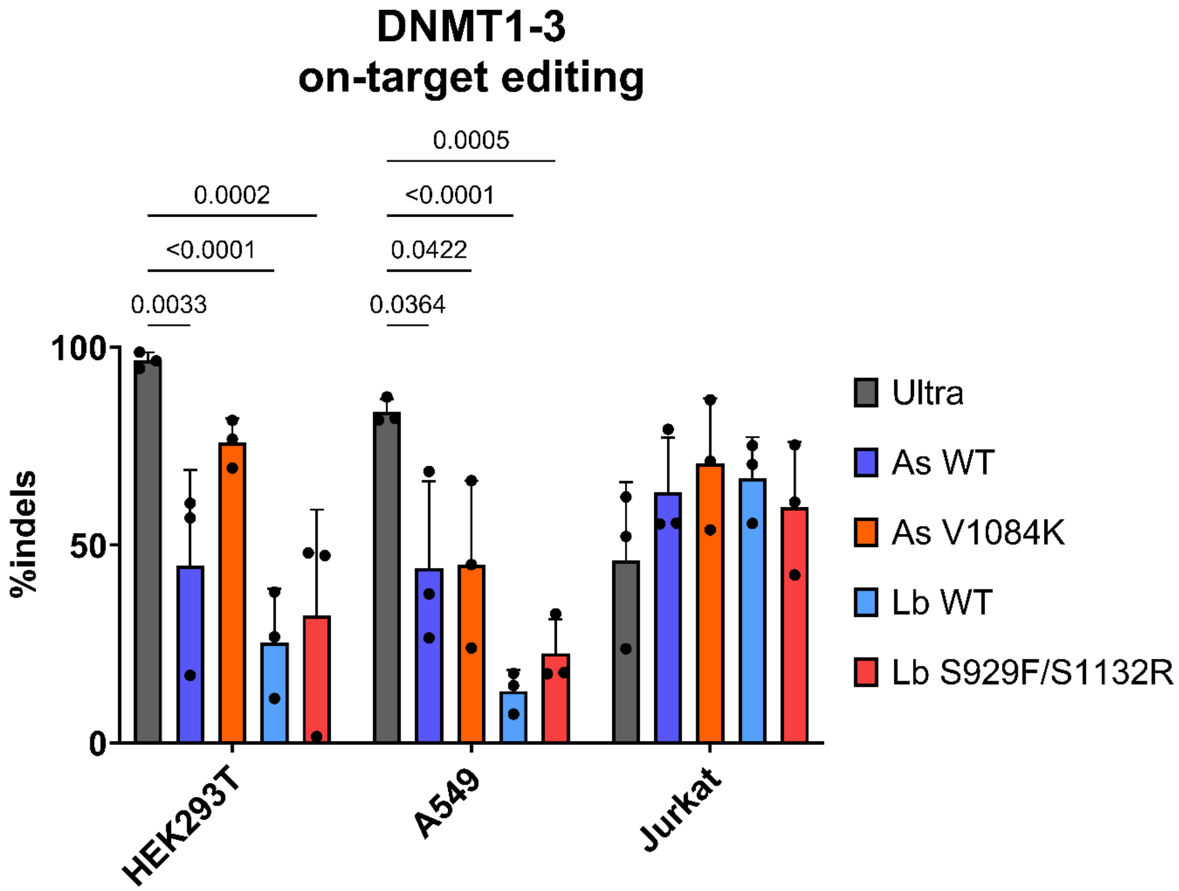

Figure S34: On-target editing with the DNMT1-3 crRNA, comparing 'Ultra' AsCas12a (IDT) vs nucleases made in this study, in the cell lines indicated on the x-axis. Bars shown mean, error bars s.d. Statistical significance evaluated by two-way ANOVA with Tukey's multiple comparison test, p-values shown for p<0.05.

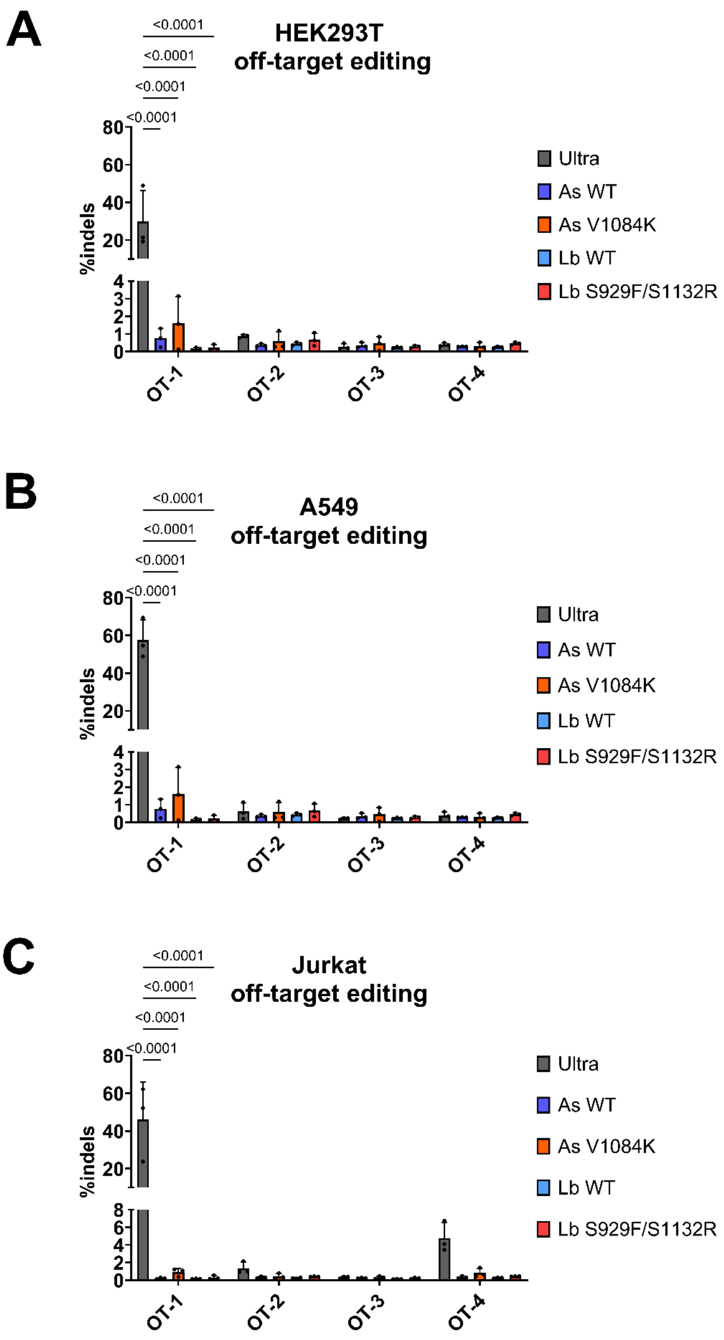

Figure S35: Off-target editing for DNMT1-3 crRNA, comparing 'Ultra' AsCas12a (IDT) vs nucleases made in this study, in (**A**) HEK293T, (**B**) A549, and (**C**) Jurkat cell lines. Bars shown mean, error bars s.d. Statistical significance evaluated by two-way ANOVA with Tukey's multiple comparison test, p-values shown for $p<0.05$.

Primer name	Sequence (5' to 3')	T°(a)
fn_f1010s_f	gatctgaactccggctttaagag	61
fn_f1010s_r	ctcgaacaccacaatggc	61
fn_k1084a_f	attcacttccgcgatctgccccgtg	62
fn_k1084a_r	ccggctggcacatagtag	62
fn_k1098a_f	gctgtaccctgcgtatgagtcagtgagcaag	63
fn_k1098a_r	tggttgacaaagccggtc	63
fn_d1135k_f	gaacttcggcaaaaaggccgcta	61
fn_d1135k_r	ttgtaatcgaaggaaaactcg	61
fn_n1212r_f	ctcagtgctgcgtacaatcctgcag	59
fn_n1212r_r	gtcagcttggcgaaaaatttc	59
fn_r1218a_f	cctgcagatggcgaactcaaagac	57
fn_r1218a_r	attgtattcagcactgag	57
as_f997s_f	aacctgaattccggctttaagagcaagag	68
as_f997s_r	ctccagcaccaccacggc	68
as_k1072a_f	atatacatctgcgatcgatcccctgaccg	64
as_k1072a_r	ggggcaggcacgtaaaac	64
as_k1086a_f	cttcgtgtgggcgaccatcaagaatcacgagag	67
as_k1086a_r	gggtccacgaagccggtc	67
as_v1084k_f	ggacccccttcaaatggaaaaccatcaagaatcacgagagc	69
as_v1084k_r	acgaagccggtcaggggga	69
as_r1220a_f	gccctgatcgcgagcgtgctgcagatgc	66
as_r1220a_r	caccatggtgtcgatggcgtgag	66
as_r1226a_f	gctgcagatggcgaactccaatgccgccac	65
as_r1226a_r	acgctgcggatcagggcc	65
lb_s929f_f	gacctgaactttggctttaagaatagccg	69
lb_s929f_r	ctccagggcgatcacggc	69
lb_k1003a_f	gctgacatccgcgatcgatccatc	58
lb_k1003a_r	caggcagggatgtaaaag	58
lb_k1017a_f	ctgctgaaaaccgcgtataccagcatcgcc	61
lb_k1017a_r	gttcacaaagccggtagatgg	61
lb_k1015a_f	gtgaacctgctggcgaccaagtatacc	60
lb_k1015a_r	aaagccggtagatggatc	60
lb_k1015a_k1017a_f	cgcgtataccagcatcgccgat	65
lb_k1015a_k1017a_r	gtcgccagcaggttcacaaagcc	65
lb_r1054a_f	gaacttctctgcgacagacgccgattac	59
lb_r1054a_r	ttatagtccagggcaaac	59
lb_r1138a_f	gctgcagatggcgaacagcatcacag	63
lb_r1138a_r	atcaggctcatcagggcc	63
lb_s1132r_f	ggccctgatgcgcctgatgctgc	70
lb_s1132r_r	ataaagctagagtagaaggccttgtcg	70

Table S1: Primers used for Cas12a mutagenesis. T°(a) indicates annealing temperature in Q5 site-directed mutagenesis protocol.

Oligo name	Sequence (5' to 3')
Trans cleavage reporter ssDNA	/56-FAM/tttttttt/ZEN/ttt/3IaBkFQ/ (IDT product codes)
TS-DNMT1-3	cccggtgtcacgccacttgacaggcgagtaacagacatggaccatcaggaaacattaacgtactgatgttaacagctgacccaataagtggcagag
NTS-DNMT1-3	ctctgccacttattgggtcagctgttaacatcagtacgttaatgtttcctgatggtccatgtctgttactcgcctgtcaagtggcgtgacaccggg
Precleaved-TS-DNMT1-3	taacagacatggaccatcaggaaacattaacgtactgatgttaacagctgacccaataagtggcagag
Precleaved-NTS-DNMNT1-3	ctctgccacttattgggtcagctgttaacatcagtacgttaatgtttcctgatggtccatgt
TS-HPV16	ctctgccacttattgggtcagctgttaacatcagtacgttaatgtttcctgatggtccatgtctgttactcgcctgtcaagtggcgtgacaccggg
NTS-HPV16	tccccatgtcgtaggtactccttaaagttagtatttttatatgtagtttctgaagtagatatggcagcacataatgacatatttgtactgcgtgtagtatcaacaacagtaacaaatagttggttaccccaaca
Lb-DNMT1-3	uaauuucuacuaaguguagaucugaugguccaugucuguuacucg
As-DNMT1-3	uaauuucuacucuuguagaucugaugguccaugucuguuacucg
Fn-DNMT1-3	uaauuucuacuguuguagaucugaugguccaugucuguuacucg
Lb-HPV16	uaauuucuacuaaguguagauugaaguagauauggcagcacauaa
As-HPV16	uaauuucuacucuuguagauugaaguagauauggcagcacauaa
Fn-HPV16	uaauuucuacuguuguagauugaaguagauauggcagcacauaa
Lb_DNMT1-7	uaauuucuacuaaguguagaugcucagcaggcaccugcctcagcu
Lb_AGBL1	uaauuucuacuaaguguagaugauugaaggaaaaguuacaaaggu
As_DNMT1-7	uaauuucuacucuuguagaugcucagcaggcaccugcctcagcu
As_AGBL1	uaauuucuacucuuguagaugauugaaggaaaaguuacaaaggu

Table S2: Oligonucleotide sequences used in *cis* and *trans* cleavage assays, DNA detection, and genome editing of human cell lines.

Cas12a	k_{NTS} (s^{-1})			k_{TS} (s^{-1})		
	**Mean**	**s.d.**	**n**	**Mean**	**s.d.**	**n**
Fn R1218A	6.30e-5	3.47e-6	3	1.83e-4	1.27e-5	3
Fn WT 200 mM NaCl	0.284	0.023	2	0.254	0.046	2
Fn WT 100 mM NaCl	0.519	0.076	2	0.261	0.039	2
Fn WT	0.318	0.006	2	0.254	0.025	2
Fn WT ~1 mM NaCl	0.233	0.003	2	0.200	0.029	2
Fn K1084A	0.179	0.045	3	0.188	0.024	3
Fn K1084A/K1098A	0.188	0.008	3	0.093	0.003	3
Fn F1010S	0.337	0.200	3	0.150	0.016	3
Fn D1135K	0.229	0.018	3	0.302	0.036	3
Fn N1212R	0.249	0.117	3	0.336	0.078	3
Fn F1010S/N1212R	0.303	0.039	3	0.104	0.002	3
Fn D1135K/N1212R	0.131	0.011	3	0.240	0.014	3
Fn F1010S/D1135K/ N1212R	1.013	0.240	3	0.232	0.011	3
As R1226A	1.99e-4	6.56e-6	3	4.22e-4	7.80e-5	3
As WT	0.060	0.008	2	0.007	9.2 e-5	2
As K1072A/K1086A/ R1220A	0.029	0.008	3	0.001	1.70 e-4	3
As F997S	0.097	0.012	2	0.006	3.0e-4	2
As V1084K	0.013	0.001	3	0.011	0.003	3
Lb R1138A	2.58e-4	9.45e-6	3	7.85e-5	5.07e-6	3
Lb WT	0.104	0.025	3	0.066	0.021	3
Lb K1003A/K1017A	0.060	0.007	3	0.040	0.004	3
Lb K1015A/R1054A	0.070	0.004	3	0.057	0.011	3
Lb K1003A/K1015A/ K1017A/R1054A	0.050	0.015	3	0.044	0.010	3
Lb S929F	0.337	0.178	3	0.108	0.024	3
Lb S1132R	0.112	0.020	3	0.177	0.015	3
Lb S929F/S1132R	0.270	0.023	3	0.091	0.003	3

Table S3: Mean, s.d., and n, of kinetic values (s^{-1}) for NTS (k_{NTS}) and TS cleavage (k_{TS}), for the Cas12a indicated
